# The molecular determinants of classical pathway complement inhibition by OspEF-related proteins of *Borrelia burgdorferi*

**DOI:** 10.1016/j.jbc.2024.107236

**Published:** 2024-03-27

**Authors:** Sheila Thomas, Anna M. Schulz, John M. Leong, Tonya N. Zeczycki, Brandon L. Garcia

**Affiliations:** 1 Department of Microbiology and Immunology, Brody School of Medicine, East Carolina University, Greenville, North Carolina, USA; 2 Department of Molecular Biology and Microbiology, Tufts School of Medicine, Tufts University, Boston, Massachusetts, USA; 3 Department of Biochemistry & Molecular Biology, Brody School of Medicine, East Carolina University, Greenville, North Carolina, USA

**Keywords:** OspEF-related proteins, ElpQ, complement system, protease inhibitor, complement C1s, HDX-MS, protein-protein interaction, Lyme disease, *Borrelia burgdorferi*

## Abstract

The complement system serves as the first line of defense against invading pathogens by promoting opsonophagocytosis and bacteriolysis. Antibody-dependent activation of complement occurs through the classical pathway and relies on the activity of initiating complement proteases of the C1 complex, C1r and C1s. The causative agent of Lyme disease, *Borrelia burgdorferi*, expresses two paralogous outer surface lipoproteins of the OspEF-related protein family, ElpB and ElpQ, that act as specific inhibitors of classical pathway activation. We have previously shown that ElpB and ElpQ bind directly to C1r and C1s with high affinity and specifically inhibit C2 and C4 cleavage by C1s. To further understand how these novel protease inhibitors function, we carried out a series of hydrogen-deuterium exchange mass spectrometry (HDX-MS) experiments using ElpQ and full-length activated C1s as a model of Elp–protease interaction. Comparison of HDX-MS profiles between unbound ElpQ and the ElpQ/C1s complex revealed a putative C1s-binding site on ElpQ. HDX-MS–guided, site-directed ElpQ mutants were generated and tested for direct binding to C1r and C1s using surface plasmon resonance. Several residues within the C-terminal region of ElpQ were identified as important for protease binding, including a single conserved tyrosine residue that was required for ElpQ- and ElpB-mediated complement inhibition. Collectively, our study identifies key molecular determinants for classical pathway protease recognition by Elp proteins. This investigation improves our understanding of the unique complement inhibitory mechanism employed by Elp proteins which serve as part of a sophisticated complement evasion system present in Lyme disease spirochetes.

The complement system is an ancient proteolytic cascade of innate immunity that consists of three canonical pathways, termed the classical, lectin, and alternative pathways (1, 2, 3, 4, 5). Bacteria have evolved various mechanisms to evade the complement system of their hosts (6, 7, 8, 9, 10). For example, *Borrelia burgdorferi*, the causative agent of Lyme disease—which is responsible for an estimated half a million infections in the United States each year (11)—expresses numerous outer surface lipoproteins that protect the bacteria from complement-mediated killing (6, 12, 13, 14, 15, 16). Borrelial anti-complement lipoproteins function by either recruitment of host complement regulators to the bacterial surface (*e.g.*, factor H) or by directly binding to complement proteins and inhibiting their activity (6, 13, 15, 16, 17, 18). Ultimately, microbial complement evasion systems function to prevent complement-mediated effector functions including clearance by anaphylatoxin release, opsonization, inflammation, priming of adaptive immune responses, and membrane attack complex formation (1, 2, 3, 4, 5).

Complement pathways are defined by how the cascade is initiated and, in all cases, are catalyzed by chymotrypsin-like serine proteases. The initiating proteases of the complement system function in the context of molecular complexes involving non-enzymatic proteins that participate in the recognition of distinct molecular patterns associated with pathogens and damaged self-tissues. At targeted surfaces, inert complement proteins are then proteolytically cleaved, releasing activated chemotactic fragments known as anaphylatoxins (*i.e.*, C3a, C5a) and components such as C3b and C4b that can form surface-associated multicomponent enzymes, known as C3 and C5 convertases (1, 2, 3, 4, 5). All three complement pathways converge at the production of C3b by C3 convertases, which act to label surfaces for C3b-mediated phagocytosis and feed a powerful self-amplification loop (1, 2, 3, 4, 5). Termination of all complement pathways occurs following cleavage of C5 and the formation of the membrane attack complex (*i.e.*, C5b-9), which upon insertion into the membrane of the target cell, leads to osmotic lysis (1, 2, 3, 4, 5).

The three pathways of the complement system differ by their mode of recognition and by their initiating proteolytic events. For example, the classical pathway (CP) initiation complex, C1, is composed of C1q that is noncovalently bound to a heterotetramer of serine proteases called C1r and C1s (*i.e.*, C1qC1r_2_C1s_2_) (1, 2, 3, 4, 5). C1q binds specifically to antibody–antigen complexes, inducing autolytic activation of zymogen C1r (19, 20). Activated C1r then specifically cleaves zymogen C1s at the Arg_437_-Ile_438_ peptide bond within the C1s scissile loop, converting C1s into a fully active enzyme (19, 20). Activated C1s can then cleave C4, by which C4b is deposited on the activated surface (21). C4b binds C2 to form the CP C3 proconvertase *(i.e*., C4bC2), followed by C1s-mediated cleavage of C2 to form the active CP C3 convertase, C4bC2b (1, 2, 3, 4, 5). In the lectin pathway, mannose-binding lectin, ficolins, and other specific collectins recognize surface-linked carbohydrates or acetyl groups (5, 21). Binding of the lectin pathway pattern recognition molecules activates the mannose-binding lectin–associated proteases on the target surface. Mannose-binding lectin–associated protease-2 shares substrate specificity with C1s and leads to the formation of identical C3 convertases as those formed by CP activation (*i.e.*, C4bC2b) (5, 21). In contrast, the alternative pathway does not require specific molecular recognition but is instead spontaneously activated at a continuously low rate via a fluid phase process known as tick-over (1, 2, 3, 4, 5). It is for this reason that host regulators, such as factor H, are required to prevent activation of the alternative pathway on endogenous cells.

We have previously shown that *B. burgdorferi* produces two distinct outer surface lipoprotein families that directly inhibit the CP by binding to the C1 complex (*i.e.*, C1qC1r_2_C1s_2_) (22). BBK32 binds with high specificity to the active site of C1r, blocking autoactivation as well as C1s cleavage (6, 23, 24, 25). In contrast, the paralogous proteins ElpQ and ElpB of the OspEF-related protein family (Erp) do not share the same inhibitory mechanism with BBK32 and instead bind with high affinity and specificity to both C1r and C1s in their activated forms (22, 26). We have shown that the inhibitory activity of ElpQ and ElpB is located within the C-terminal domains of each protein-covering residues 181 to 343 and 182 to 378, respectively, and that each protein acts as a direct inhibitor of C1s in cleavage of its two native substrates C2 and C4 (26). In this study, we investigated the solution structure of the ElpQ–C1s complex using hydrogen-deuterium exchange mass spectrometry (HDX-MS) and utilized HDX-MS-guided, site-directed mutagenesis to reveal key molecular determinants involved in the unique complement inhibitory mechanism of Elp proteins.

## Results

### Analysis of the ElpQ solution structure by HDX-MS and AlphaFold2

**Figure 1:**
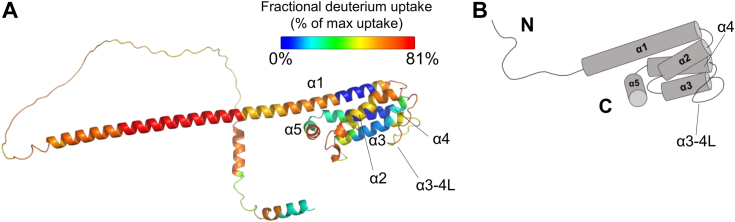
**Analysis of the solution structure of ElpQ by HDX-MS.***A*, fractional deuterium uptake of full-length Apo-ElpQ_19-343_ mapped onto an AlphaFold2 model, UniProt: Q9S035. Exposure at 120 min. Values are shown as a spectrum gradient of % of maximum uptake ranging from 0 to 81%. Alpha-helices 1 thru 5 are numbered and labeled: alpha-helix (α); long loop connecting alpha-helices 3 and 4 (α3-4L). HDX reactions were performed in triplicate (Apo-ElpQ_19-343_). *B*, topology of the predicted ElpQ structure. N- and C-terminal regions are labeled as N or C, respectively. HDX-MS, hydrogen-deuterium exchange mass spectrometry.

To better understand the interactions formed in solution between full-length ElpQ and full-length human C1s, we carried out a series of HDX-MS experiments (Figs. S1–S3 and Tables S1–S4). Each sample was incubated in a deuterium solvent and deuteriation was monitored across five timepoints out to 2 h. A total common sequence coverage of 96% was achieved involving 110 unique peptides across the 325 residue ElpQ protein (Table S1). While an experimental three-dimensional structure of ElpQ has not been reported, an AlphaFold2 model predicts the presence of a disordered N-terminal region linked to a C-terminal alpha-helical bundle (Fig. S4). This model agrees with our previous far UV-circular dichroism data that showed full-length and C-terminally truncated ElpQ proteins predominantly adopted an alpha-helical secondary structure (26). We mapped the fractional deuterium uptake following a 2 h exchange for Apo-ElpQ_19-343_ onto this model (Fig. 1). Consistent with the AlphaFold2 prediction, deuterium uptake is lowest within the C-terminal alpha-helical bundle and highest for N-terminal residues, although we noted that maximal uptake is observed for the predicted extended N-terminal region of α-helix 1, suggesting that this region is likely disordered in solution (Fig. 1). A similar fractional uptake pattern for the C-terminal region of a truncation construct of ElpQ (*i.e.*, ElpQ_181-343_) was also observed, suggesting that the absence of the disordered N-terminal region of ElpQ does not dramatically alter the solution structure of the C-terminal domain of ElpQ (Fig. S5*A*).

### Mapping of C1s-binding sites on full-length ElpQ using HDX-MS

We have previously shown that ElpQ preferentially binds to the activated form of purified human C1s with a *K*_D_ value of ∼5 nM, assuming a 1:1 model of binding (22). We have biochemically mapped this interaction to the C-terminal region of ElpQ (*i.e.*, ElpQ_181-343_); however, the specific residues involved in C1s-binding are unknown. Peptides with altered HDX profiles in the ElpQ-C1s sample relative to the unbound Apo-ElpQ_19-343_ sample can provide information on possible C1s-binding sites. To identify ElpQ residues involved in C1s complex formation, we monitored deuterium uptake of ElpQ over time (0–120 min) in the absence or presence of a 3-fold molar excess of full-length activated C1s (Table S1).

**Figure 2:**
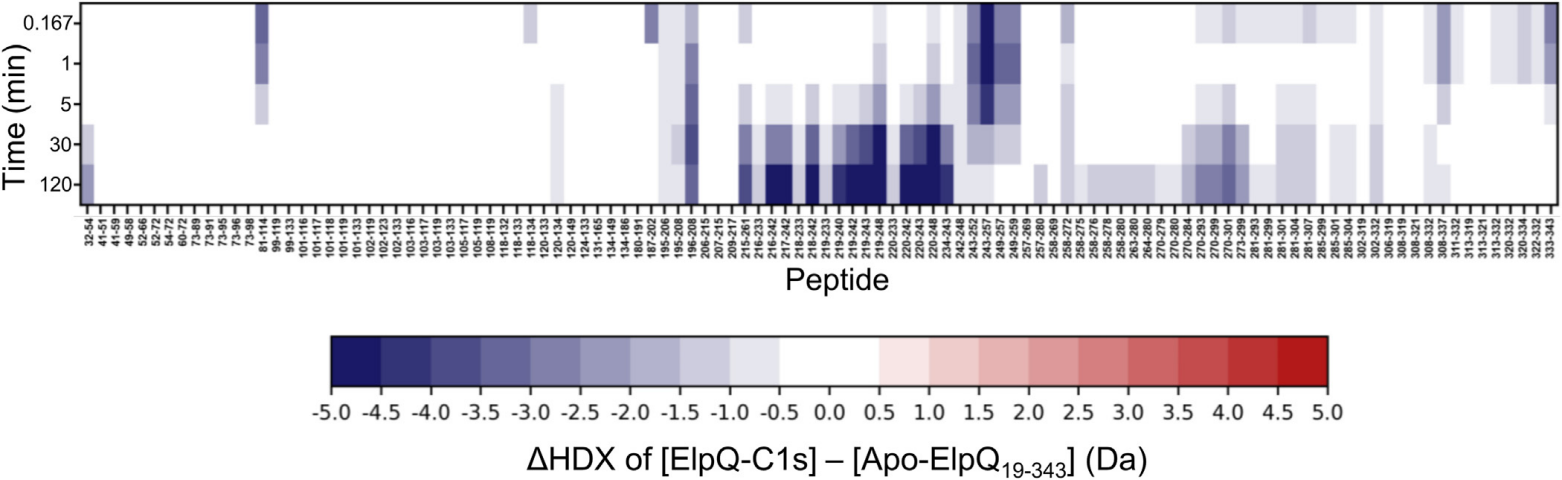
**Global analysis of differential uptake (ΔHDX) between ElpQ-C1s and Apo-ElpQ**_**19-343**_**.** A chiclet plot was produced using HD-eXplosion showing the average deuterium uptake difference ΔHDX = [ElpQ-C1s] (bound) - [Apo-ElpQ_19-343_] (unbound) in Daltons (Da). Time (min) is plotted over all statistically significant peptides (*p-*value <0.01; n = 3). Values are shown as a *blue*-*white*-*red* gradient with the lowest values in *dark blue* and highest values shown in *red*. *White* indicates no difference between the two datasets.

Differential deuterium uptake between the ElpQ-C1s and Apo-ElpQ_19-343_ samples (ΔHDX) was first analyzed by plotting the average deuterium uptake difference in the form of a ‘chiclet’ plot using HD-eXplosion (27, 28, 29, 30) (Fig. 2). Chiclet plots allow for the simultaneous visualization of ΔHDX values across all experimental timepoints (27, 28, 29, 30). Negative values (blue) correspond to the protection of ElpQ from HDX in the presence of C1s and positive values (red) correspond to deprotection in the presence of C1s. Many of the peptides exhibited statistically significant negative ΔHDX values (*p*-value < 0.01, Welch’s *t* test); these peptides were almost exclusively concentrated between residues 180 to 343 (Fig. 2). These results suggest that specific residues from the C-terminal region of ElpQ form a binding interface with C1s, which is consistent with our previous analysis showing that a C-terminal truncation mutant (*i.e.*, ElpQ_181-343_) bound to C1s with similar affinity to full-length ElpQ (26).

Further analysis of the data presented in Figure 2 allowed for a more detailed view of the potential ElpQ/C1s-binding interface. While all peptides exhibited EX2 type exchange, the nature of interacting peptides could be subdivided into three types: (i) peptides covering residues 195 to 208 and 270 to 304 are protected from HDX at most timepoints, while in contrast, (ii) peptides covering residues 243 to 259 and residues 313 to 343 are initially protected from exchange, but over time reach equal uptake, and finally (iii) reduced ΔHDX values for peptides covering residues 215 to 243 are only observed at later timepoints, which is a unique pattern in the plot. Collectively, these data suggest that the interaction between ElpQ and C1s is localized to the C-terminal region of ElpQ that are under different HDX kinetic regimes.

**Figure 3:**
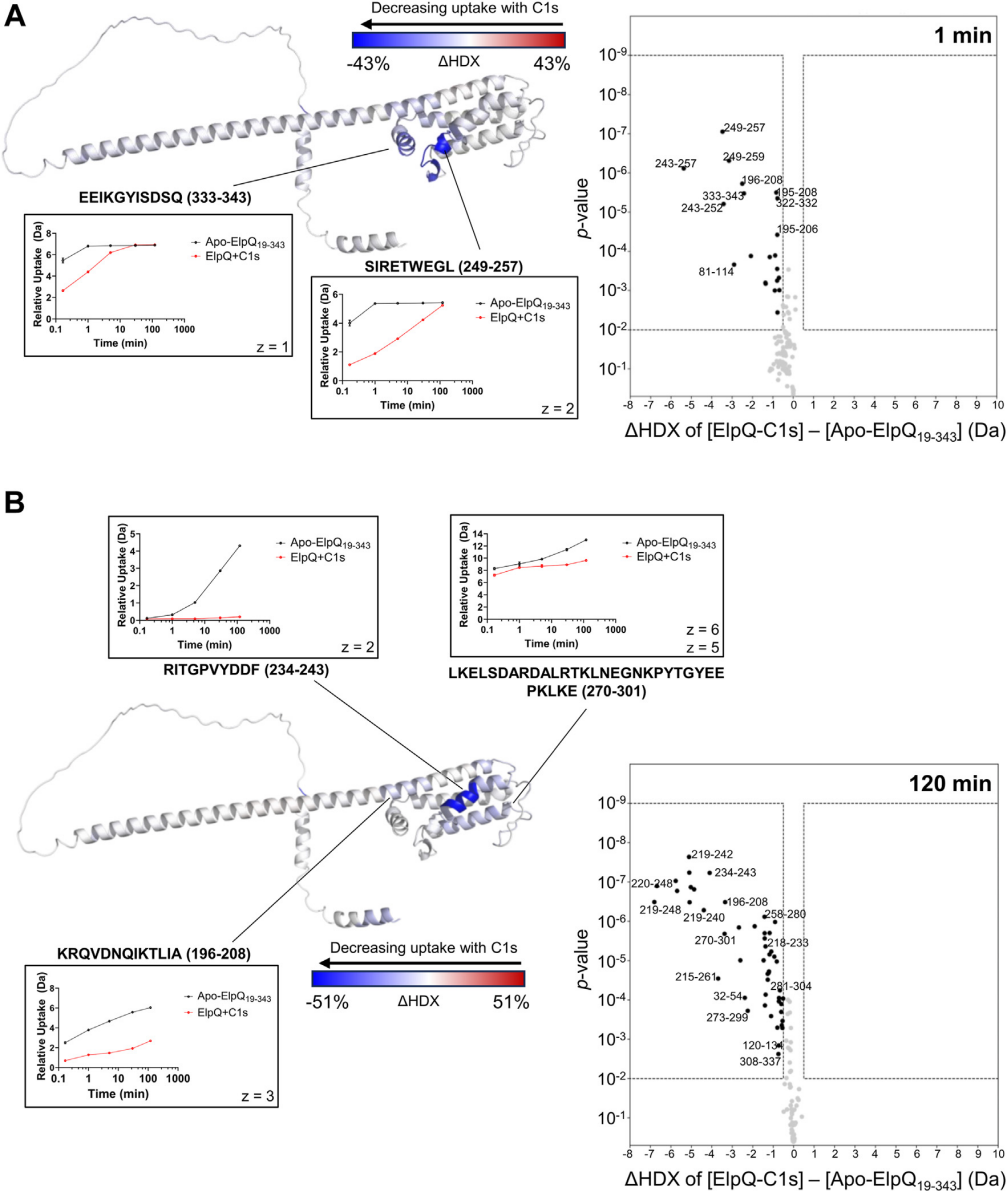
**HDX-MS mapping of C1s-binding sites on full-length ElpQ.** Volcano plot of average ΔHDX determined by subtracting [Apo-ElpQ_19-343_] from [ElpQ-C1s] at (*A*) 1 min or (*B*) 120 min. The difference in uptake is plotted over the *p*-value calculated using Welch’s *t* test. Each *dot* represents a single peptide. *Black dots* are labeled and indicate statistically significant peptides (n = 3). *Dots* within *dashed boxes* show peptides with *p*-value < 0.01 and a minimum absolute average uptake difference set to 0.5 Da. Uptake plots from representative example peptides are shown with relative fractional uptake plotted over time in min. *Red* indicates ElpQ-C1s, and *black* is Apo-ElpQ_19-343_. Charge states for each peptide are indicated as z. Peptides were mapped onto the AlphaFold2 model of ElpQ using the DynamX per residue output without statistics and PyMOL. A *blue*-*white*-*red* gradient indicates ΔHDX as % of maximum. HDX-MS, hydrogen-deuterium exchange mass spectrometry.

To visualize the potential C1s interaction surfaces on ElpQ, we projected ΔHDX values onto the ElpQ Alphafold2 model using DynamX v3.0 for a timepoint early in the experiment (*i.e.*, 1 min) and the final timepoint (*i.e.*, 120 min) (Fig. 3). Representative uptake plots are shown for peptides exhibiting significant differences in deuterium uptake between the ElpQ-C1s sample and the Apo-ElpQ_19-343_ sample as judged by volcano plot analyses using HD-eXplosion. Uptake plots are presented as the mean uptake from three separate injections at each timepoint with error bars showing the SD. All associated data including SD values are shown in Table S2. This analysis reveals that residues 196 to 208, 249 to 257, and 334 to 343 appear to form a molecular surface on one side of the C-terminal helical bundle that participates in interactions with C1s (Figs. 3*A* and S1*B*). Additionally, residues between 234 to 243 on alpha-helix 2 (α2) and 270 to 301 (α3 and α3-4L), along with 196 to 208 (α1), also present a surface that forms stable interactions with C1s (Figs. 3*B* and S1*B*). In this regard, residues 234 to 243 are interesting in that deuterium uptake in the Apo-ElpQ_19-343_ is consistent with a region exhibiting slow dynamics. Initially, this region is protected from exchange in the unbound form of the protein, potentially due to extensive hydrogen-bonding of the amide hydrogens of 234 to 243 (31, 32, 33). Over time, hydrogen-bond separation allows residues to take up deuterium which is observed at a later time point (Fig. 3*B*, inset, black line). However, in the presence of C1s, HDX is completely blocked at this site even at later time points, thus movement is potentially restricted (Fig. 3*B*, inset, red line) (31, 34).

### Molecular determinants for C1s binding

**Figure 4:**
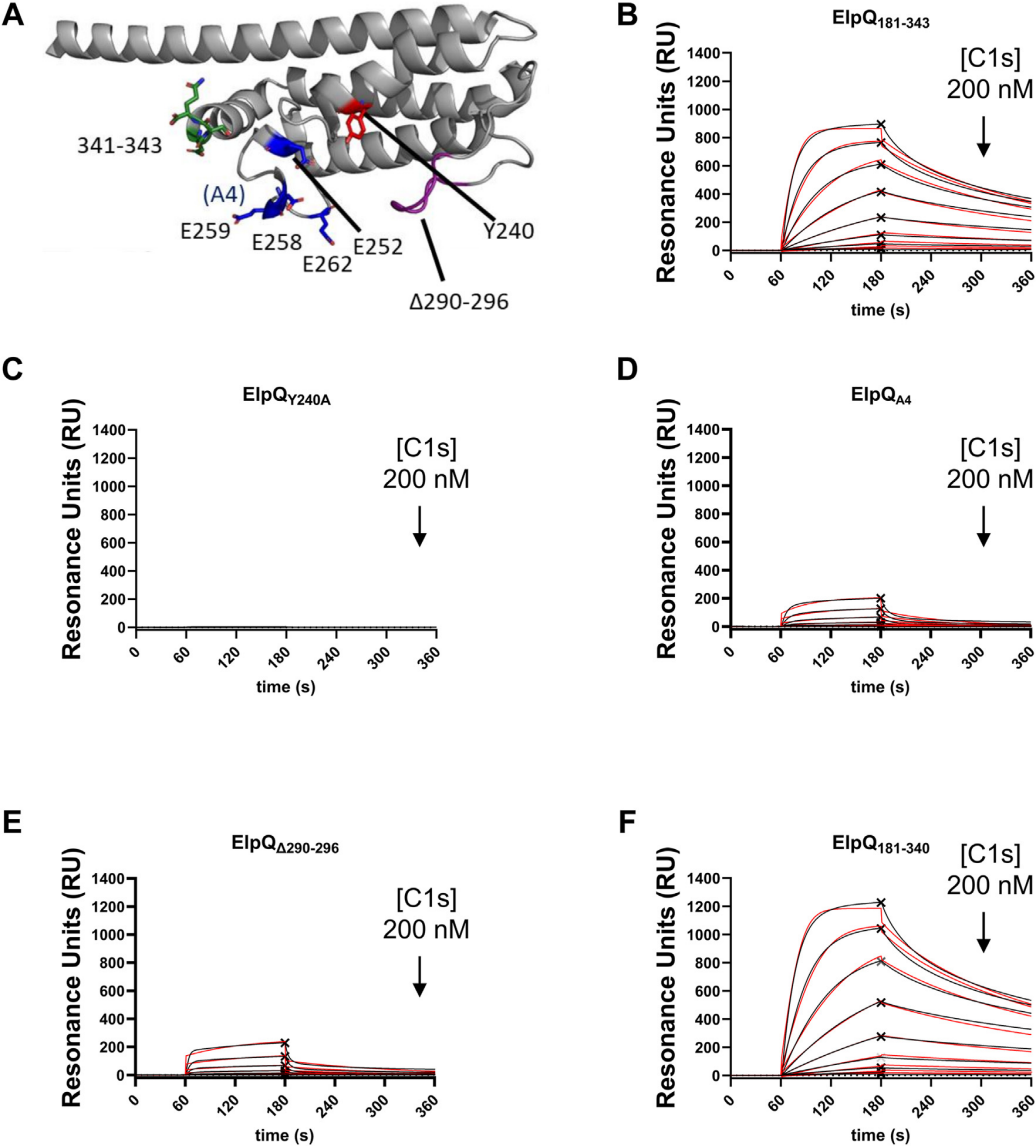
**Identification of ElpQ residues that mediate C1s-binding.***A*, site-directed mutants were generated in the background of ElpQ_181-343_ (*gray*) based on HDX-MS analysis. Mutants involved (i) a single substitution of tyrosine for alanine (ElpQ_Y240A_, *red*), (ii) four clustered glutamates (*i.e.*, E252, E258, E259, and E262) substituted for alanine residues (ElpQ_A4_, *blue*), (iii) deletions of residues 290 to 296 (ElpQ_Δ290-296_, *purple*), and iv) an ElpQ truncation lacking residues 341 to 343 (ElpQ_181-340_, *green*). ElpQ site-directed mutant proteins were immobilized on an SPR sensor chip, and activated human C1s was injected over the biosensors across a two-fold dilution series ranging from 0 to 200 nM. Sensorgrams from a representative injection series are shown. *B*, ElpQ_181-343_. *C*, ElpQ_Y240A_. *D*, ElpQ_A4_. *E*, ElpQ_Δ290-296_. *F*, ElpQ_181-340_. Sensorgrams are shown as *black lines* and *red lines* indicate kinetic fits. The X symbol represents steady-state response. Each injection series was performed in triplicate. Kinetic (*K*_D, kin_) and steady-state (*K*_D, ss_) fits of the reference- and background-corrected sensorgrams were determined using a 1:1 binding model (Langmuir). SDs were calculated from triplicate injection series on two separate days (ElpQ_181-343_, ElpQ_A4,_ or ElpQ_181-340_; n = 6) or on 1 day (ElpQ_Δ290-296_ or ElpQ_Y240_; n = 3). HDX-MS, hydrogen-deuterium exchange mass spectrometry.

Analysis of the HDX-MS experiments described above suggested that specific residues within the C-terminal region of ElpQ are involved in mediating ElpQ/C1s protein–protein interactions. To validate this, we generated four site-directed ElpQ mutants involving residues originating from peptides exhibiting significant differences in deuterium uptake between the ElpQ-C1s and Apo-ElpQ_19-343_ samples (Fig. 4*A*). All mutated proteins were created in the background of the ElpQ_181-343_ truncation which has previously been shown to bind directly to C1s (26), a result that is further supported by the HDX-MS mapping experiments (Figs. 2 and 3). The first mutation was introduced into the dynamic 234 to 243 region by generating a single alanine substitution at Y240 (*i.e.*, ElpQ_Y240A_) and because the AlphaFold2 model predicts Y240 to be surface exposed. The second mutated protein (*i.e.*, ElpQ_A4_) targeted four glutamate residues with alanine substitutions that are predicted to be closely arranged within the central region of the helical bundle, presenting a negatively charged surface (*i.e.*, residues 252–262). The next mutation targeted the long loop connecting α3 and α4 with a truncation (*i.e.*, ElpQ_Δ290-296_). The last mutation targeted the predicted disordered region of the C-terminal cap, residues 341 to 343, via truncation of these three residues (*i.e.*, ElpQ_181-340_). Importantly, CD experiments confirmed that each site-directed mutant, including the truncation mutants, retained a WT-like alpha-helical fold (Fig. S6) (26).

We first tested the ability of these mutated proteins to bind to C1s relative to ElpQ_181-343_ using surface plasmon resonance (SPR) by immobilizing each protein onto an SPR sensor chip. A concentration series ranging between 0.78 and 200 nM of purified full-length activated C1s was injected over each ElpQ mutant biosensor and compared to ElpQ_181-343_ (Fig. 4, *B*–*F*). The resulting sensorgrams were used to calculate equilibrium dissociation constants (*K*_D_) using both kinetic analysis (*K*_D, kin_) and steady-state analysis (*K*_D, ss_) (Table 1). The control surface of ElpQ_181-343_ exhibited a *K*_D, kin_ value of 14.5 nM and *K*_D, ss_ value of 39.3 nM (Table 1), which agrees well with our previously published values of 17 nM and 31 nM, respectively (26). While ElpQ_181-340_ exhibited *K*_D_ values similar to WT (Fig. 4*F*), ElpQ_A4_ had a ∼10-fold reduction in binding affinity and ElpQ_Δ290-296_ had a ∼15-20-fold reduction (Fig. 4, *D* and *E*). Interestingly, ElpQ_Y240A_ failed to produce a measurable binding response at the concentrations used in the assay and *K*_D_ values could not be determined (Fig. 4*C*). These observations suggest that key residues near the central region of the ElpQ C-terminal helical bundle are involved in C1s binding, and Y240 is essential for a high-affinity interaction.

**Table 1:** SPR binding parameters

SPR	C1s					C1r				
Ligands	*k*_a_ (1/Ms)	*k*_d_ (1/s)	*K*_D, kin_ (nM)	*K*_D, ss_ (nM)	R_max_ (RU)	*k*_a_ (1/Ms)	*k*_d_ (1/s)	*K*_D, kin_ (nM)	*K*_D, ss_ (nM)	R_max_ (RU)
ElpQ_181-343_	4.77 × 10^5^ (3.69 × 10^4^)	6.83 × 10^−3^ (6.23 × 10^−4^)	14.5 (2.42)	39.3 (1.03)	748 (162)	2.17 × 10^4^ (4.22 × 10^3^)	3.16 × 10^−3^ (1.20 × 10^−4^)	151 (34.6)	221 (18.4)	433 (28.6)
ElpQ_Y240A_	NB	NB	NB	NB	NB	NB	NB	NB	NB	NB
ElpQ_A4_	6.01 × 10^4^ (1.64 × 10^4^)	1.03 × 10^−2^ (2.29 × 10^−3^)	173 (11.0)	464 (95.2)^a^	189 (10.7)	2.44 × 10^3^ (3.02 × 10^3^)	4.93 × 10^−1^ (1.06 × 10^0^)	ND	ND	82.5 (30.7)
ElpQ_Δ290-296_	2.25 × 10^4^ (1.51 × 10^4^)	7.46 × 10^−3^ (3.32 × 10^−4^)	623 (655)^a^	665 (40.3)^a^	227 (1.76)	NB	NB	NB	NB	NB
ElpQ_181-340_	4.25 × 10^5^ (1.20 × 10^5^)	7.94 × 10^−3^ (1.41 × 10^−3^)	20.8 (9.21)	55.6 (10.6)	830 (436)	2.64 × 10^4^ (5.08 × 10^3^)	2.77 × 10^−3^ (7.21 × 10^−4^)	113 (48.2)	181 (81.9)	557 (226)
ElpB_Y266A_	NB	NB	NB	NB	NB	NB	NB	NB	NB	NB

Association rate constants are shown as *k*_a_ or dissociation rate constants, *k*_d_. Equilibrium dissociation constants (*K*_D_) were calculated by performing kinetic (*K*_D, kin_) or steady-state analysis (*K*_D, ss_) for each interaction, using a 1:1 binding model. Values in parentheses indicate SD (See Experimental procedures).

a *K*_D_ was outside of range of tested concentrations and is thus an estimate. R_max_, maximal binding response; ND, not determined since experimental curves did not fit 1:1 binding model; NB, no measurable binding detected.

### Molecular determinants for C1r binding

**Figure 5:**
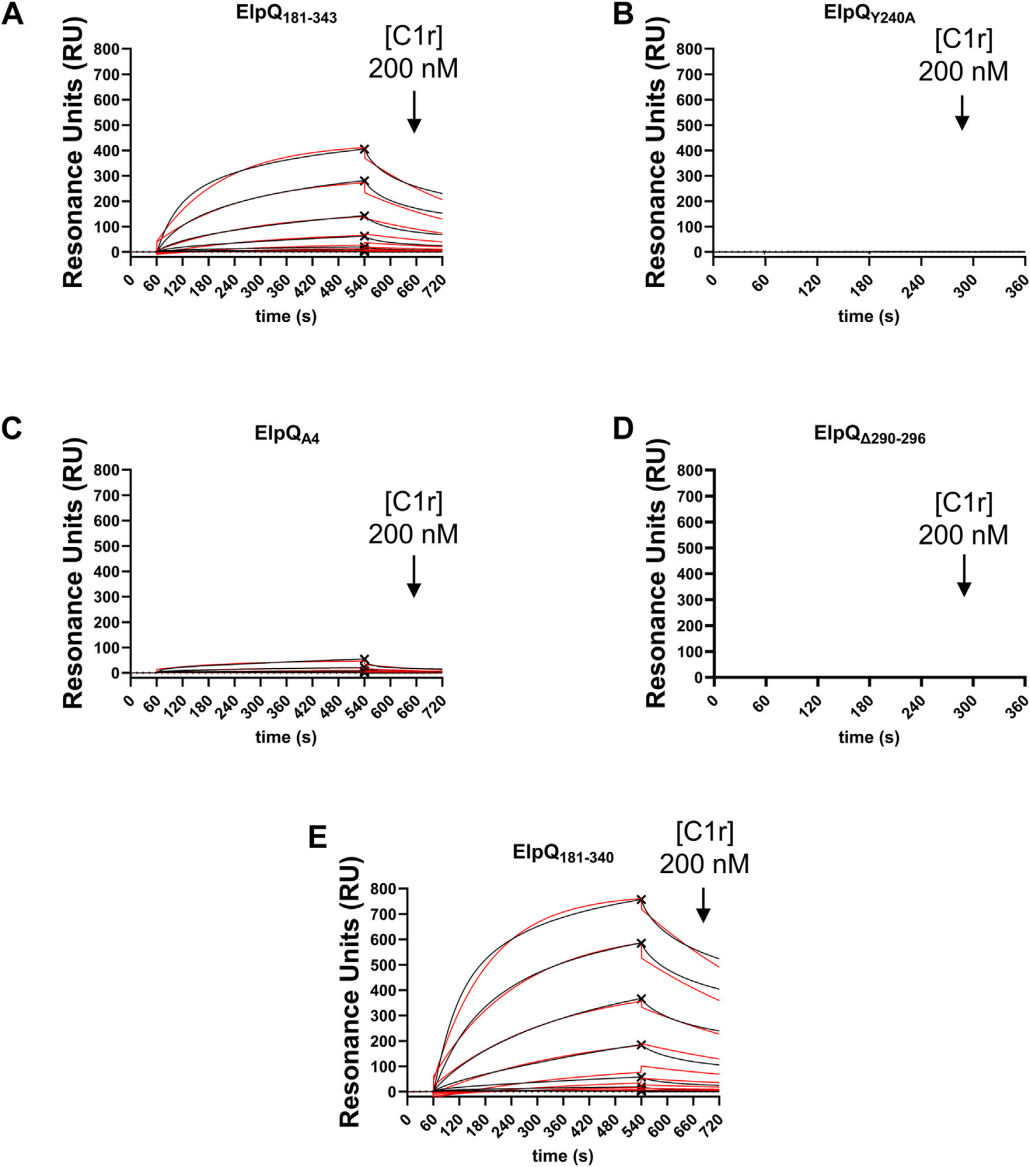
**Identification of ElpQ residues that mediate C1r-binding.** ElpQ site-directed mutant proteins were immobilized on an SPR sensor chip, and activated human C1r was injected over the biosensors across a two-fold dilution series from 0 to 200 nM. Sensorgrams from a representative injection series are shown. *A*, ElpQ_181-343_. *B*, ElpQ_Y240A_. *C*, ElpQ_A4_. *D*, ElpQ_Δ290-296_. *E*, ElpQ_181-340_. Sensorgrams are shown as *black lines* and *red lines* indicate kinetic fit. The X symbol represents steady-state fit. Each injection was performed in triplicate. Kinetic (*K*_D, kin_) and steady-state (*K*_D, ss_) fits of the reference- and background-corrected sensorgrams were determined using a 1:1 binding model (Langmuir). SDs were calculated from triplicate injection series on two separate days (ElpQ_181-343_, ElpQ_A4,_ or ElpQ_181-340_; n = 6) or on 1 day (ElpQ_Δ290-296_ or ElpQ_Y240_; n = 3).

While the inhibitory mechanism of ElpQ appears to be at the level of C1s (22, 26), we have previously shown that ElpQ also recognizes C1r with high affinity. As little is known about the role of ElpQ/C1r-binding, we wondered if the C1s-binding mutants identified from HDX-MS analysis of the ElpQ/C1s complex also impacted ElpQ/C1r binding. Using SPR, activated full-length human C1r was injected in a dose-dependent manner over ElpQ_181-343_ and each ElpQ mutant (Fig. 5). ElpQ_181-343_ exhibited a calculated *K*_D, ss_ value of 221 nM, which is comparable to the previously obtained *K*_D, ss_ value of 97 nM for a full-length ElpQ GST fusion protein (22). This suggests that like C1s, the C1r-binding site for ElpQ is located on the C-terminal helical bundle. Furthermore, the ElpQ_181-340_ mutant bound to C1r with WT-like affinity (*K*_D, ss_ = 181 nM) (Fig. 5*E*) and the binding to C1r by ElpQ_Y240A_ could not be detected (Fig. 5*B*), mirroring the observations for C1s binding. Interestingly, a relatively weak dose-dependent binding of C1r by ElpQ_A4_ was observed, and the data could not be fit to a 1:1 binding kinetic model, and steady-state affinity analysis produced *K*_D_ values far outside the concentration range of C1r used in the assay (Fig. 5*C*). Surprisingly, ElpQ_Δ290-296_ failed to produce a measurable binding signal at the concentrations used, suggesting these residues play a more critical role in C1r recognition relative to C1s (Fig. 5*D*). Collectively, these results suggest that while there is at least a partially overlapping binding site on ElpQ for both proteases, there are differences in the relative role of the glutamate residues mutated in the ElpQ_A4_ construct and the loop residues missing in ElpQ_Δ290-296_, in binding to C1r versus C1s.

### HDX-MS-guided, site-directed ElpQ mutants are impaired for complement inhibitory activity

**Figure 6:**
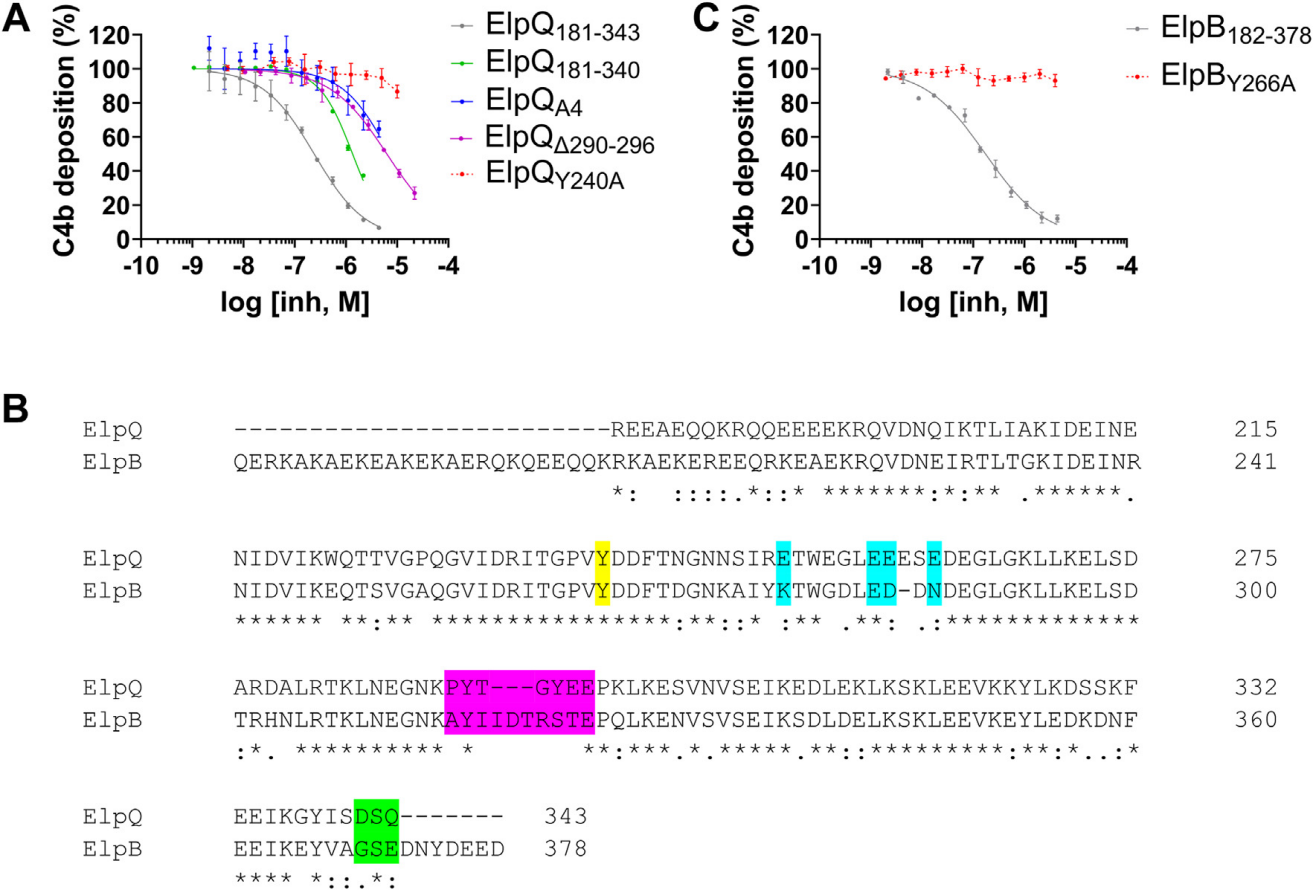
**A conserved tyrosine residue is important for classical pathway inhibitory activity in ElpQ and ElpB.***A*, CP ELISA assays for ElpQ mutants were performed using C4b deposition as a marker for complement activation. The inhibitory activity of a concentration series of each protein was evaluated by non-linear regression. *Dashed lines* indicate data could not be fitted. Assays were repeated in triplicate and error bars depict SD. *B*, amino acid sequence alignment of the C-terminal regions of *Borrelia burgdorferi* strain B31 ElpB (residues 182–378) and ElpQ (residues 181–343). Symbols indicate the following: (∗) identical, (:) strongly similar, (.) weakly similar, (−) missing residue. Site-directed ElpQ mutants are highlighted as follows: Y240 (*yellow*), A4 (*blue*), Δ290 to 296 (*purple*), and truncation of last three residues (*green*). *C*, CP ELISA assays for ElpB_182-378_ and ElpB_Y266A_. IC_50_ values were obtained by nonlinear regression analysis using a four-parameter variable slope fit and constraining the *bottom* and *top* values to 0 and 100, respectively. The 95% confidence interval (95% CI) values are shown in Table 2. CP, classical pathway.

Next, we tested whether the ElpQ mutants discussed above have altered CP inhibition properties in a serum-based CP activation ELISA (Fig. 6*A*). ElpQ_181-343_ exhibited a half-maximal inhibitory concentration (IC_50_) of 240 nM, which is similar to the previously reported value of 210 nM (Table 2) (26). By comparison, all four mutants inhibited CP-mediated C4b deposition with weaker IC_50_ values with the potency rank order of ElpQ_181-340_ (IC_50_ = 1350 nM) > ElpQ_Δ290-296_ (IC_50_ = 6310 nM) > ElpQ_A4_ (IC_50_ = 7120 nM) > ElpQ_Y240A_ (IC_50_ = not determined) (Fig. 6*A*, Table 2). Thus, the inhibitory potency correlates well with C1s affinity and to a lesser extent, C1r affinity (Table 1).

**Table 2:** CP ELISA potencies

CP ELISA	ElpQ_181-343_	ElpQ_Y240A_	ElpQ_A4_	ElpQ_Δ290-296_	ElpQ_181-340_	ElpB_182-378_	ElpB_Y266A_
IC_50_ (nM)	240	ND	7120	6310	1350	175	ND
95% CI (nM)	209–277	ND	4660–15,000	5770–6920	1280–1430	156–195	ND

IC_50_ values were obtained by nonlinear regression analysis using a four-parameter variable slope fit and constraining the *bottom* and *top* values to 0 and 100, respectively. The 95% confidence interval (95% CI) is shown. ND, not determined.

### A conserved tyrosine mediates complement inhibition in ElpB

We have previously demonstrated that the C-terminal region of the ElpQ paralog, ElpB (residues 182–378), also interacts with activated forms of C1s and C1r and inhibits the CP with similar affinity and potency to ElpQ (22, 26). HDX-MS experiments using Apo-ElpB_182-378_ showed that it exhibits a similar fractional uptake profile compared to Apo-ElpQ_181-343_, which is consistent with the similarity in folds predicted by AlphaFold2 (Fig. S5*B*). A sequence alignment revealed that of the four HDX-MS-guided ElpQ mutants tested here, only residues from ElpQ 234 to 243 (*i.e.*, RITGPVYDDF) are 100% conserved between the two proteins, including the key ElpQ-Y240 residue (Fig. 6*B*). We mutated this residue in the truncated form of ElpB (*i.e.*, ElpB_Y266A_) and compared its activity to WT in a CP ELISA assay. Just as was seen for ElpQ_Y240A_, mutation of Y266 in ElpB produced a protein with no measurable inhibition of CP-mediated C4b deposition (Fig. 6*C*, Table 2). As expected from this result, we observed no detectable response from this mutant in either C1s or C1r in SPR-binding assays (Fig. S7), similar to our results for ElpQ_Y240A_. Taken together, these data show that the ElpQ residues identified in the HDX-MS mapping experiments are important for mediating complement inhibition and that Y240—which is conserved in ElpB—is indispensable for this activity in both proteins.

## Discussion

**Figure 7:**
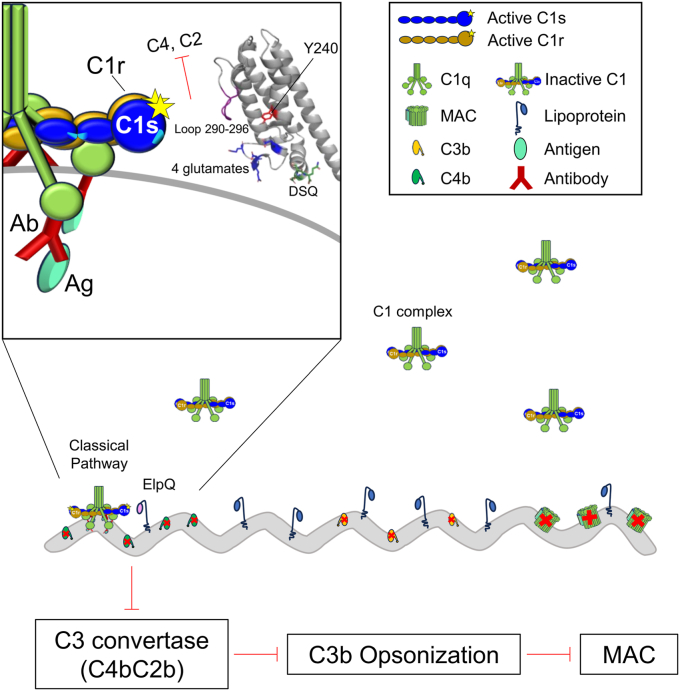
**Model of the ElpQ–C1s interaction impeding downstream complement-mediated killing of *Borrelia burgdorferi*.** Activation of the classical pathway begins at the C1 complex. In the unbound state, a heterotetramer composed of C1s_2_ and C1r_2_ are present in their zymogen form and in complex with C1q. C1q recognizes and binds to antibodies bound to borrelial antigens on the bacterial surface (*gray*). C1q bound to the immune complex then triggers proteolytic autoactivation of C1r (*brown*, starred), which then cleaves and activates C1s (*blue*, starred). The surface lipoprotein *B. burgdorferi* ElpQ interacts with active C1s and C1r via interactions from the long loop region covering residues 290 to 296 (*purple*), four glutamates on a smaller loop connecting alpha-helices 2 and 3 (*royal blue*), residues DSQ of the C-terminal cap, and a hot-spot interaction involving tyrosine 240. On C1s, the bacterial protein binds to two C4-exosites located between domains CCP1 and CCP2 (site highlighted in *light blue*, *left*, and *right*, respectively) and on the serine protease domain (site in *light blue*, far *right*) (26). This binding event blocks C1s from cleaving C4 and C2, resulting in the inhibition of C3 convertase formation which indirectly prevents downstream C3 cleavage and C3b deposition, anaphylatoxin release, and the assembly of the membrane attack complex (MAC).

HDX-MS can be utilized to determine binding sites, conformational changes, and dynamic properties of proteins (35). Previously, HDX-MS has been used to map epitopes of monoclonal antibodies to *B. burgdorferi* OspA (36), but to our knowledge, this technique has not been used to evaluate interactions formed between native host proteins and borrelial outer surface lipoproteins. Here, we utilized HDX-MS to probe the ElpQ/C1s protein–protein interface and validated this analysis using HDX-MS-guided, site-directed mutagenesis. This approach led to the identification of several key amino acids in ElpQ that are important for its complement inhibitory activity (Fig. 7). Thus, HDX-MS may serve as a tractable approach to investigate the underlying molecular mechanisms for the plethora of host-pathogen protein–protein interactions that contribute to the pathogenesis of Lyme disease or other microbial pathogens.

Mapping of the HDX-MS data onto the three-dimensional AlphaFold2 model of ElpQ suggests that a contiguous C1s-binding surface is formed on one face of the C-terminal alpha-helical bundle of ElpQ. More specifically, site-directed mutants indicate that Y240 on the second alpha-helix (*i.e.*, ElpQ_Y240A_), a cluster of four glutamate residues on the loop connecting the second and third helix (*i.e.*, ElpQ_A4_), a loop structure that connects alpha-helices 3 and 4 (*i.e.*, ElpQ_Δ290-296_), and three residues positioned on the C-terminal alpha-helical “cap” (*i.e.*, ElpQ_181-340_), all contribute to C1s binding. In particular, ElpQ_Y240A_, ElpQ_Δ290-296_, and ElpQ_A4_ mutants exhibited a dramatic reduction in both C1s affinity and inhibitory potency (Tables 1 and 2). Despite having a modest effect on C1s affinity, ElpQ_181-340_ exhibited a ∼6-fold reduction in inhibitory potency (Table 2), suggesting these residues may play a role in the underlying inhibitory mechanism. While we acknowledge that it is likely that additional residues play roles in driving C1r and C1s affinity than those tested here, the loss-of-function mutants identified in this study may be leveraged in future studies investigating the role of C1-mediated inhibition by ElpQ on the spirochetal surface.

Our previous work has shown that ElpQ interacts with C4-exosites on C1s that are presumably absent in C1r (26). Despite this, ElpQ binds to C1r with high affinity (22). Here, we found that ElpQ/C1s HDX-MS-guided, site directed mutants were also deficient in binding to activated C1r (Table 1). Strikingly, Y240 is required for binding both C1r and C1s (Figs. 4 and 5, Table 1). However, we note that ElpQ_Δ290-296_ and ElpQ_A4_ had a more profound reduction in C1r binding than C1s (Table 1). This result suggests that ElpQ harbors a site that is shared between C1s and C1r (*i.e.*, Y240) and other sites that exhibit binding preference to C1r. Given that C1s-binding affinity correlates strongly with ElpQ inhibitory potency (Tables 1 and 2) and our previous linking of C1s proteolytic inhibition to its complement inhibitory activities (26), future studies will be needed to more fully address the potential role of C1r interaction in Elp function.

Our previous reports support the notion that the complement inhibitory activities of ElpB are functionally redundant to ElpQ (22, 26). The identification here of a conserved tyrosine that is required for potent CP inhibition by both ElpQ and ElpB further suggests that these two proteins share inhibitory mechanisms. However, analysis of the fractional deuterium uptake between Apo-ElpQ and Apo-ElpB proteins revealed potential differences in the dynamics of peptides harboring ElpB-Y266 (Figs. 1 and S5). In Apo-ElpQ_19-343_, residues 235 to 242 exhibit low deuterium uptake at 1 min before increasing to 54% at 120 min, suggesting a dynamic region of the apo-protein (Figs. 1 and 3*B*, S5*A*). This phenomenon was also seen at the 30 min timepoint of the Apo-ElpQ_181-343_ construct (Figs. 1 and S5*A*). However, in complex with C1s, this site remains protected at 120 min and only exhibits 3% relative fractional uptake (Fig. 3*B*). Investigating the conformational dynamics of this key structural element and how this contributes to the function of the broader family of Elp proteins is an important question for future studies.

The paralogous ElpB and ElpQ proteins are members of the wider OspEF-related proteins (Erps) but are distinguished from other Erps based on their larger molecular weight and associated differences in three dimensional protein structures (37, 38, 39, 40, 41, 42). Like other Erps, ElpB and ElpQ expression is induced during tick feeding and persists during mammalian infection (37, 41, 43, 44, 45). In addition to ElpB and ElpQ, *B. burgdorferi* strain B31 encodes for the production of three additional paralogs (22, 37, 38, 46). Interestingly, residues homologous to ElpQ-Y240 are conserved in all *B. burgdorferi* strain B31 Elp paralogs and this residue is also conserved in the related proteins from strains N40 and 297 (Fig. S8). However, other residues shown here to be important in CP inhibition by ElpQ (*i.e.*, residues mutated in ElpQ_A4_, ElpQ_Δ290-296_, and ElpQ_181-340_) are less conserved in both identity and sequence length (Fig. S8). One potential explanation for the presence of a large paralogous family of CP inhibitors with plasticity in their host protease–binding sites may be related to previous work with other complement-binding borrelial lipoproteins that differentially recognize complement proteins across vertebrate species (17, 47, 48, 49, 50, 51, 52, 53, 54, 55). For example, the factor H-binding proteins CspZ and CspA have host species-specific–binding properties that are related to sequence variation in their respective factor H-binding domains (17, 47, 48, 49, 50, 51, 52). Whether different Elp proteins also exhibit host-specific complement inhibition activities is an important question to be addressed by future studies.

In summary, using HDX-MS, we have identified several key amino acids that serve as molecular determinants for the specific interaction of ElpQ with the classical pathway complement proteases, C1r and C1s. Our investigation shows that specific residues within the central alpha-helical bundle are critical for driving CP inhibition in both ElpQ and ElpB. These results improve our understanding of an unusual class of borrelial protease inhibitors and expand our knowledge on the molecular mechanisms that are employed by human microbial pathogens to evade the complement system.

## Experimental procedures

### Bacterial plasmids, strains, and culture conditions

ElpQ site-directed mutants were generated in the background of the “wild-type” ElpQ_181-343_ truncation construct and ElpB_Y266A_ in the “wild-type” ElpB_182-378_ truncation construct. DNA fragments corresponding to each mutant gene were *Escherichia coli* codon optimized and synthetically produced by Integrated DNA Technologies gBlock Gene Fragment service with 5′BamHI and 3′NotI sites. The only exception was the ElpQ_181-340_ construct, which was PCR-amplified with encoded 5′BamHI and 3′NotI restriction sites from the previously generated pT7HMT-ElpQ_181-343_ plasmid using Q5 Master Mix (NEB) as previously described (23, 26, 56). All DNA fragments were subjected to enzyme restriction digestion, ligated into pT7HMT, and then transformed into *E. coli* DH5α as previously described (23, 26, 56). Transformants were selected using LB media plates supplemented with kanamycin (50 μg/ml), and sequences of each insert were verified following plasmid isolation (Eurofins).

### Protein expression and purification of recombinant proteins

All recombinant ElpQ and ElpB proteins were produced in *E. coli* BL21(DE3) and purified using methods previously described (22, 26). Purified activated human C1r and C1s proteins were purchased from Complement Technologies.

### CD spectroscopy

Far UV-CD was utilized for secondary structure determination of truncated or site-directed mutants of ElpQ proteins and ElpB_Y266A_, using previously described methods (26). Briefly, samples were buffer exchanged into 10 mM of Na_3_PO_4_. ElpQ samples were diluted to 10 μM and ElpB_Y266A_ was diluted to 19.3 μM. Spectra were collected using Chirascan V100 (Applied Photophysics). Samples were measured using a square quartz cuvette with a path length of 0.05 cm or 1 mm across a wavelength 180 to 300 nm, at 120 nm/min, using 1 nm step, 0.5 s response, and 1 nm bandwidth. Spectra were background corrected against the matching buffer and calculated as molar ellipticity (deg × cm^2^/dmol) using Pro-Data Viewer (v 4.8.3.313, Applied Photophysics).

### ELISA-based classical pathway inhibition assay

CP ELISA assays were carried out as previously described (26). Briefly, human IgM (3 μg/ml) (MP Biomedical) in coating buffer (100 mM Na_2_CO_3_/NaHCO_3_ [pH 9.6]) was immobilized on a high-binding ELISA plate (Greiner Bio-One). Dose-dependent ElpQ or ElpB inhibition was determined using a 12-point, two-fold dilution: ElpQ_181-343_ (4.4–0.002 μM), ElpQ_Y240A_ (10–0.005 μM), ElpQ_A4_ (4.4–0.002 μM), ElpQ_Δ290-296_ (22–0.011 μM), ElpQ_181-340_ (2.2–0.001 μM), ElpQ_182-378_ (4.4–0.002 μM), or ElpB_Y266A_ (4.0–0.002 μM). Normal human serum (2%, Innovative Research) was added, along with either ElpQ or ElpB inhibitor in CP buffer (10 mM Hepes [pH 7.3], 0.1% [w/v] gelatin, 140 mM NaCl, 2 mM CaCl_2_, 0.5 mM MgCl_2_). All assays were performed in triplicate and normalized to positive (100%, no inhibitor) and negative (0%, no normal human serum) controls. Inhibition curves were fitted using nonlinear regression to a normalized variable slope model in GraphPad Prism (v 10.0.2, GraphPad Software, Inc) (www.graphpad.com).

### Hydrogen-deuterium exchange mass spectrometry

#### HDX-MS experiments

Local amide HDX experiments were carried out using a fully automated system (PAL, LEAP Technologies). HDX reactions for Apo-ElpQ_19-343_ (10 μM), Apo-ElpQ_181-343_ (20 μM), or Apo-ElpB_182-378_ (20 μM) were initiated by mixing with a 19-fold (v/v) excess of D_2_O (99.8% D_2_O)-containing exchange buffer (57 μl, 25 mM phosphate buffer [pD 7.5], 0.1 M NaCl). After incubation for 10 s to 2 h (Apo-ElpQ_19-343_) or 10 s to 30 min (Apo-ElpQ_181-343_ or Apo-ElpB_182-378_), unwanted back exchange was minimized with the addition of 1 equivalent (v/v, 60 μl) of cold quench solution (200 mM phosphate buffer [pH 2.5], 0 °C). For unlabeled reactions (*e.g.* “0 s exchange”), apo-proteins were mixed with a 20-fold excess of H_2_O-containing buffer (25 mM phosphate buffer [pH 7.5], 0.1 M NaCl) and quenched as above.

To determine the binding site of C1s, differential HDX for ElpQ was determined in a 3-fold molar excess of C1s. Proteins were incubated on ice for ∼20 min prior to analysis. Exchange reactions were monitored at 0 s to 2 h, at 20 °C, quenched with quench solution, and digested as above. After quenching, all samples (100 μl) were immediately loaded onto an Enzymate BEH Pepsin Column (2.1 × 30 mm, 5 μm). Digestion temperature was maintained at 12 °C, a flow rate of 250 μl/min, and a pressure of ∼8000 psi for 4 min. Peptides (10–15 pmol) were subjected to LC/MS-MS analysis immediately following digestion. To help prevent sample carry-over between injections, the inline pepsin column was washed (1.5 M GuHCl, 4% acetonitrile, 0.8% formic acid [pH 2.5]) after each sample digestion, and clean blank injections (0.1% formic acid) were run after every protein injection. HDX reactions were performed in triplicate (Apo-ElpQ_19-343_ or ElpQ-C1s) or duplicate (Apo-ElpQ_181-343_ or Apo-ElpB_182-378_).

#### LC/MS-MS

LC-MS/MS analysis was performed using a Synapt XS mass spectrometer in positive mode (Waters) coupled to a nano-Acquity UPLC/HDX manager system (Waters). Post-pepsin digested products were directly loaded onto an Acquity UPLC BEH C18 Vanguard trapping column (1 × 5 mm, 250 μl/min) and desalted for 4 min. Peptides were separated on a Acquity UPLC BEH C18 1.7 μm analytical column (1 × 50 mm) using an effective 7-min linear gradient starting from 5% acetonitrile, 0.1% formic acid to 60% acetonitrile, 0.1% formic acid at a flow rate of 50 μl/min. All chromatographic steps, including trapping and elution, were performed at 2 °C. HDMS^E^ data were collected with an ESI capillary voltage of 3.2 kV and the quadrupole used in rf-mode; only ions with m/z >300 were transmitted. The collision energy in the trap was continuously alternated between low energy (4 V) and high energy (20–35 V) throughout the run. For all measurements, ToF were acquired in resolution mode with a scan time of 0.4 s. Data were lock-mass corrected post-acquisition using the 1+ charge state of LeuEnk [MH^+^ 556.2771], which was infused at a concentration of 200 pg/μl at 90° to the analytical sprayer at 10 μl/min throughout the acquisition.

#### Peptide identification and data processing

Data were processed using ProteinLynx Global Server (PLGS v 3.03, Waters). Data were centroided, de-isotoped, and charge state reduced prior to fragment ion and parent protein assignments based on retention time alignments. For peak picking, 25 counts and 100 counts were used for all high and low energies. Peak lists were searched against databases containing the either (Uniprot Q9S035) or (Uniprot H7C7R2) and porcine pepsin (Uniprot P00791). Protein identification criteria were set as the detection of at least three fragments per protein, three fragments per peptide, and one peptide per protein. Methionine oxidation was set as a variable modification for all searches. The protein level false discovery rate was set to 4%. PLGS search results and deuterium exchange spectra were imported into DynamX (v 3.0, Waters), and peptides were filtered based on the following criteria: ElpQ: three fragments per peptide, two fragments must be adjacent, have a minimum of 0.2 products per amino acid residue or ElpB: three fragments per peptide, one fragment must be adjacent, have a minimum of 0.3 products per amino acid residue and be present in all of the LC/MS-MS injections. Deuterium exchange measurements were analyzed with default settings and data were manually inspected, validated, and curated. In keeping with recommended reporting standards (35) for HDX-MS experiments, HDX summary data files, peptide coverage maps, and all uptake plots are provided (Figs. S1–S3 and Tables S1–S4). For all data sets, differences in fractional deuterium uptake over time or between conditions were mapped onto AF-Q9S035-F1-model_v4 (ElpQ) and AF-H7C7R2-F1-model_v4 (ElpB) using scripts generated in DynamX and PyMOL (https://www.schrodinger.com/platform/products/pymol/). Heat maps were generated from DynamX software (www.waters.com/waters/library.htm?locale=en_US&lid=134832928), where exposure data is rendered from the shortest peptide and peptides with residues closest to the C-terminus. The software does not render exposure data from the N-terminus of each peptide. Average HDX-MS data was obtained from State Data generated in DynamX. HDX-MS data analysis was carried out using the web-based application HD-eXplosion (27, 28, 29, 30). The *p*-value was set to less than 0.01 and was calculated using a two-tailed Welch’s *t* test. A minimum absolute average uptake difference was set to 0.5 Da.

### Protein structure modeling

Structure predictions of ElpQ and ElpB were obtained from the AlphaFold2 database (https://alphafold.ebi.ac.uk/) (42) and all depictions of protein structure were produced using PyMOL (The PyMOL Molecular Graphics System, v 2.5.4, Schrödinger, LLC).

### Surface plasmon resonance

General methods for performing SPR-binding assays were used as previously described (22, 23, 26). ElpQ protein truncations and site-directed mutants were amine coupled to a CMD200 sensor chip (XanTec bioanalytics) using 10 μg/ml in 10 mM sodium acetate (pH 4.0) with final immobilization densities, measured in resonance units (RU), as follows: ElpQ_181-343_ (1997.2 RU), ElpQ_Y240A_ (651.5 RU), ElpQ_A4_ (2137.5 RU), ElpQ_Δ290-296_ (796.1 RU), ElpQ_181-340_ (4043.3 RU), or ElpB_Y266A_ (101.1 RU). All assays were carried out in running buffer of HBS-T Ca^2+^ (10 mM Hepes [pH 7.3], 140 mM NaCl, 0.005% [v/v] Tween-20, and 5 mM CaCl_2_) with a flow rate of 30 μl/min. Analytes were exchanged into matching running buffer prior to experimentation and after each analyte injections; surfaces were regenerated to baseline using three 60 s injections of 2 M NaCl. ElpQ proteins and the ElpB mutant interacting with C1s or C1r were evaluated using multicycle experiments with active C1s or C1r (Complement Technology), with an injection series (0, 0.8, 1.6, 3.1, 6.3, 12.5, 25, 50, 100, and 200 nM) over an association time of 120 s (for C1r, 480 s) and a dissociation time of 180 s. All injection series were performed in triplicate. Kinetic (*K*_D, kin_) and steady-state (*K*_D, ss_) fits of the reference- and background-corrected sensorgrams were determined using a 1:1 binding model (Langmuir) using Biacore T200 Evaluation Software (www.cytivalifesciences.com/en/us/support/software/biacore-downloads) (v 3.1, Cytiva). SDs were calculated from triplicate injection series on two separate days (ElpQ_181-343_, ElpQ_A4,_ or ElpQ_181-340_; n = 6) or on 1 day (ElpQ_Δ290-296_, ElpQ_Y240_, or ElpB_Y266A_; n = 3).

### Alignment

Full-length amino acid sequences of ElpQ, ElpB, and Elp homologs were aligned using Clustal Omega. Uniprot numbers, ElpD_B31 (Q44791); ElpB_B31 (H7C7R2); ElpQ_B31 (Q9S035); ElpX_B31 (H7C7L6); ElpM_B31 (H7C7M1); Elp22_N40 (Q8GL37); Elp24_N40 (Q8GL34); Elp26_N40 (Q8GL33); ElpA1_297 (Q9X3N9); ElpA2_297 (Q9X3P0); ElpB1_297 (O87298); ElpB2_297 (O87306).

## Data availability

All data are contained within the article.

## Supporting information

This article contains supporting information.

## Conflict of interest

The authors declare that they have no conflicts of interest with the contents of this article..
