# Supplementary material for: The molecular determinants of classical pathway complement inhibition by OspEF-related proteins of Borrelia burgdorferi
Source: J Biol Chem. 2024 Mar 27;300(5):107236. doi: 10.1016/j.jbc.2024.107236 (PMC11066524; doi:10.1016/j.jbc.2024.107236)
Supplement: Supporting Information [file mmc1.docx]

The molecular determinants of classical pathway complement inhibition by OspEF-related proteins of *Borrelia burgdorferi*

Sheila Thomas^1^, Anna M. Schulz^1^, John M. Leong^2^, Tonya N. Zeczycki^3^, and Brandon L. Garcia^1*^

Running Title: ElpQ HDX-MS

^1^Department of Microbiology and Immunology, Brody School of Medicine, East Carolina University, Greenville, North Carolina, USA

^2^Department of Molecular Biology and Microbiology, Tufts School of Medicine, Tufts University, Boston, Massachusetts, USA

^3^Department of Biochemistry & Molecular Biology, Brody School of Medicine, East Carolina University, Greenville, North Carolina, USA

*** Correspondence:**Brandon L. Garcia
garciabr18@ecu.edu

**This file contains Figures S1-S8; Table S1.**


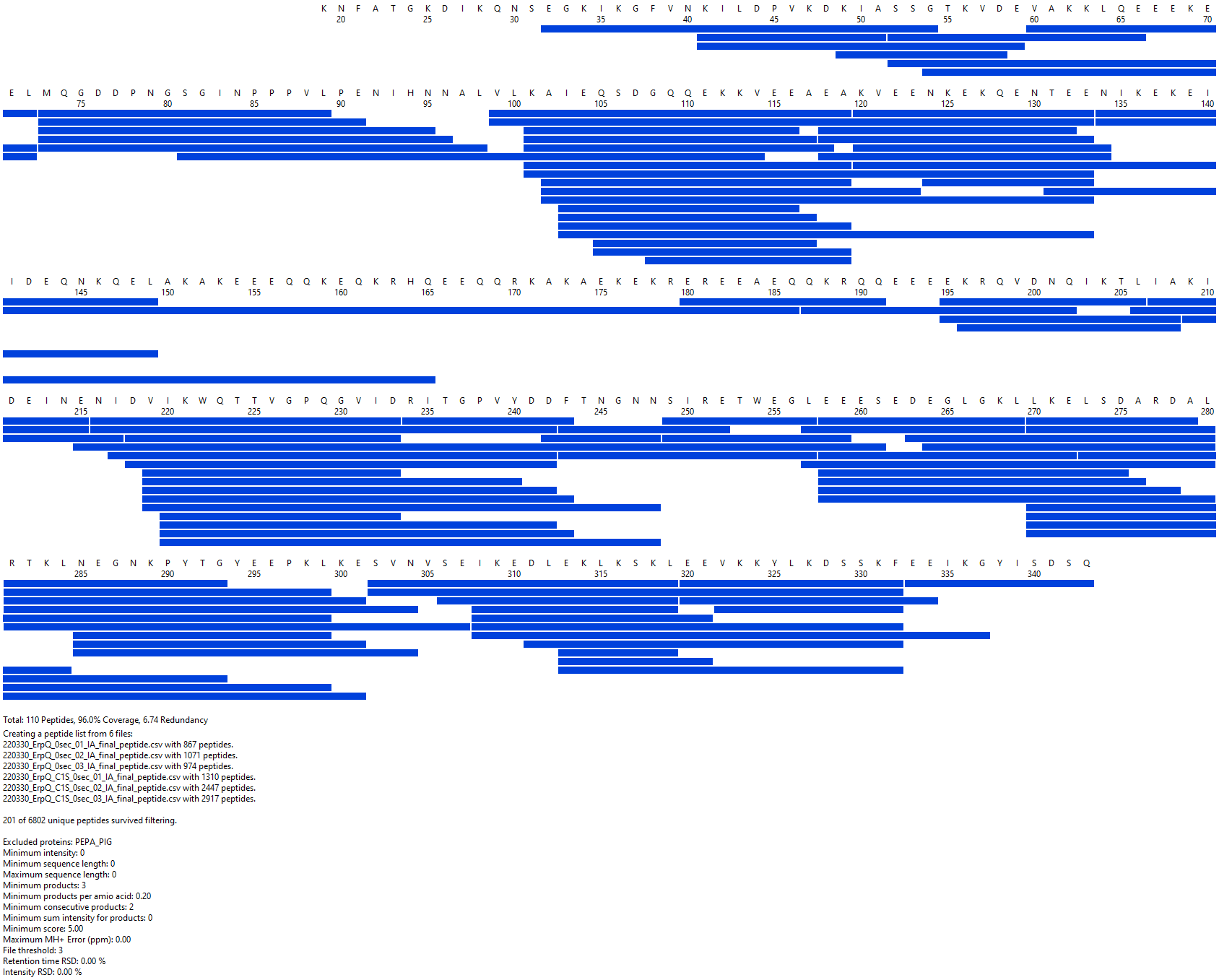


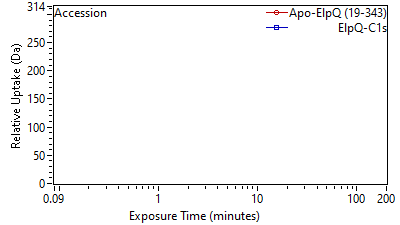

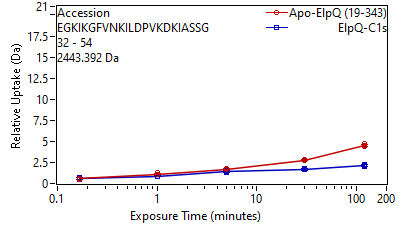

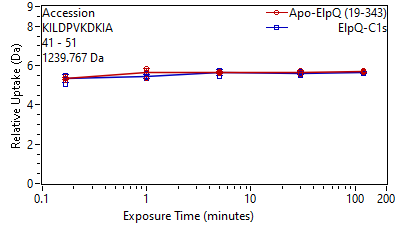

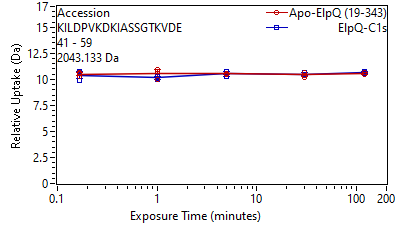

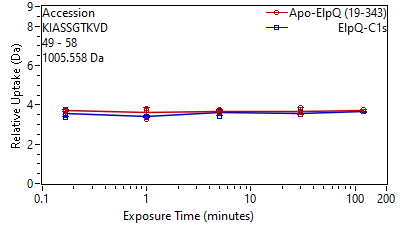

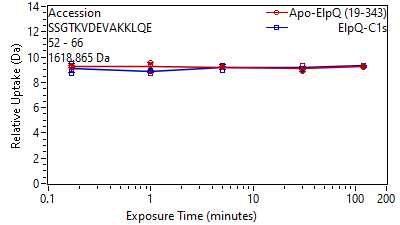

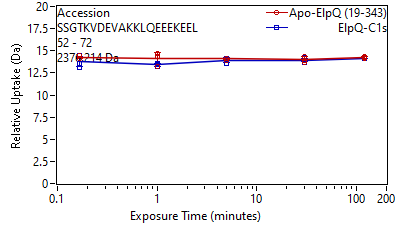

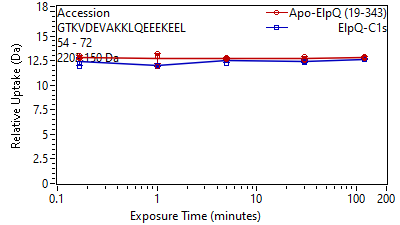

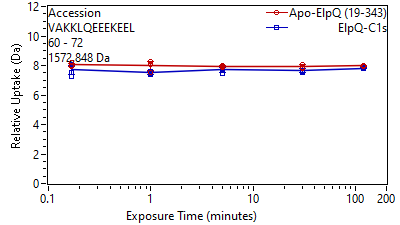

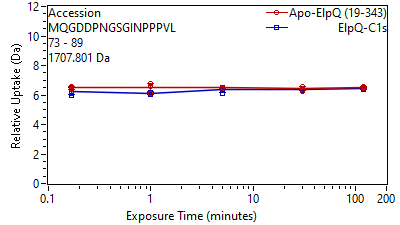

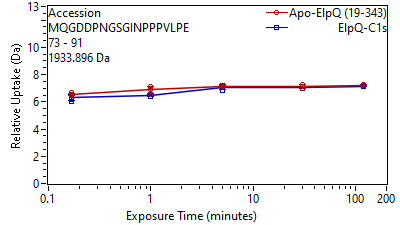

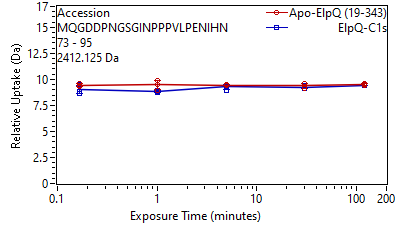

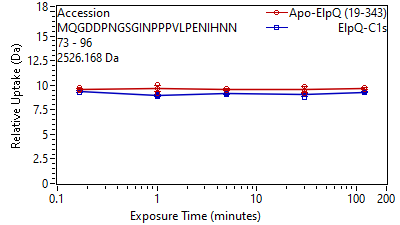

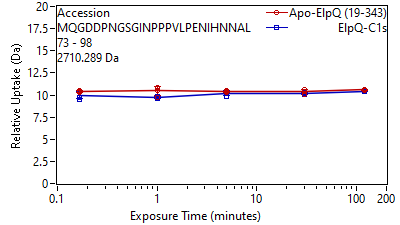

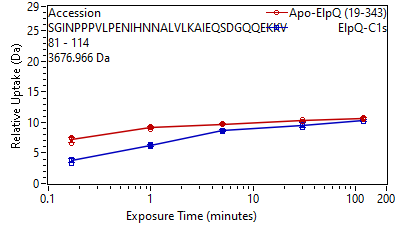

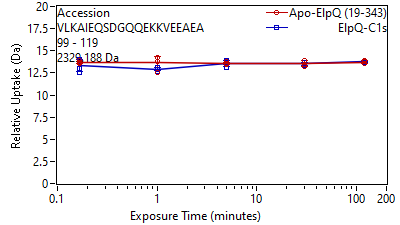

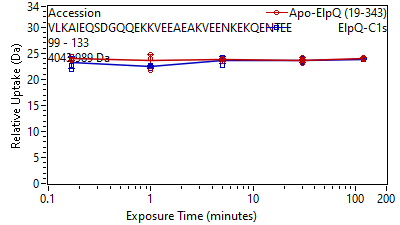

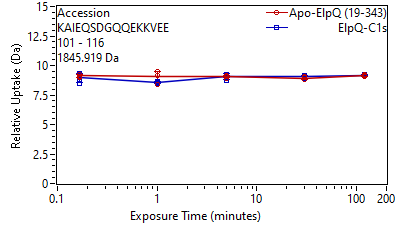

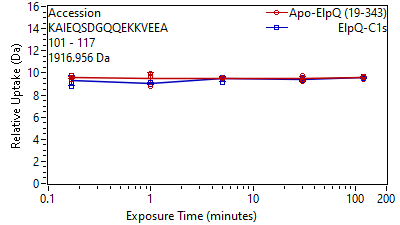

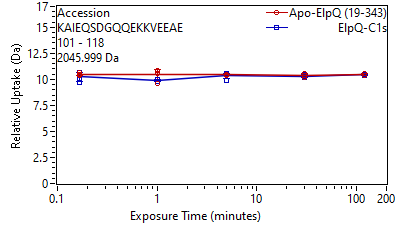

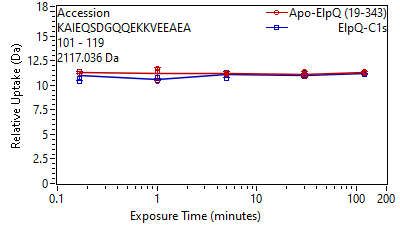

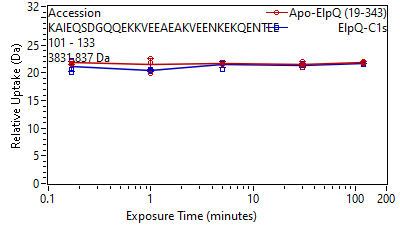

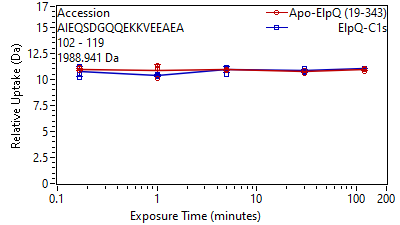

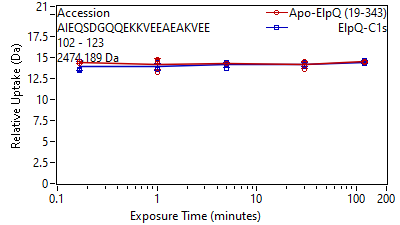

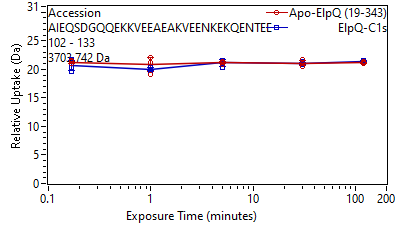

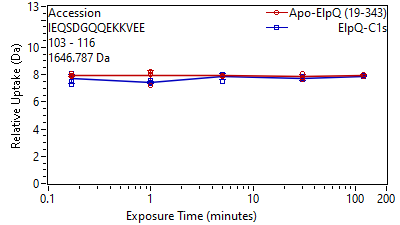

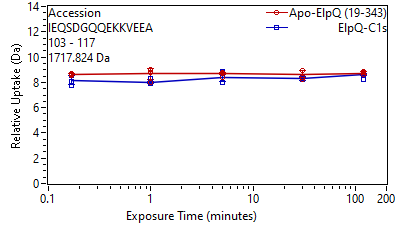

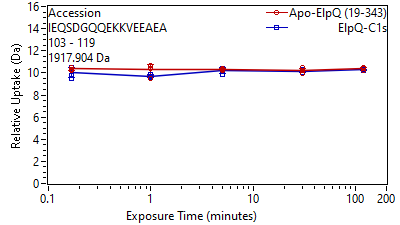

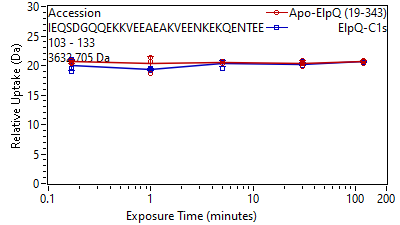

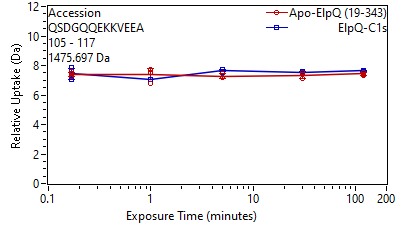

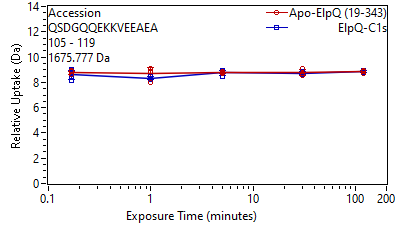

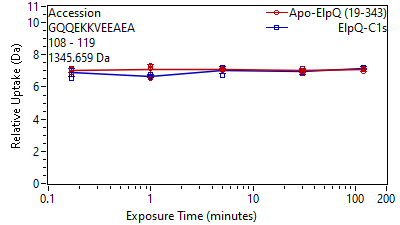

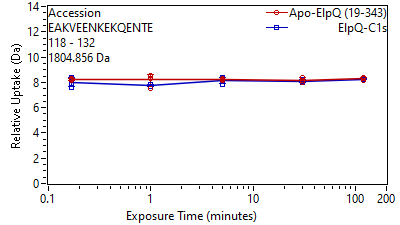

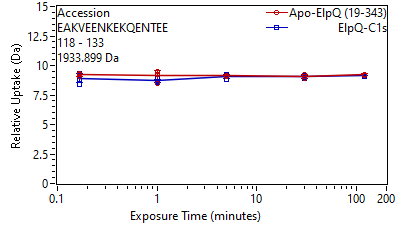

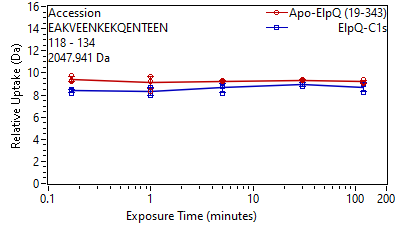

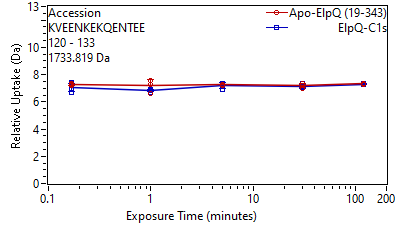

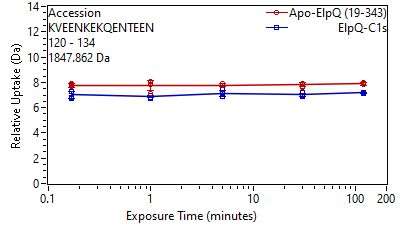

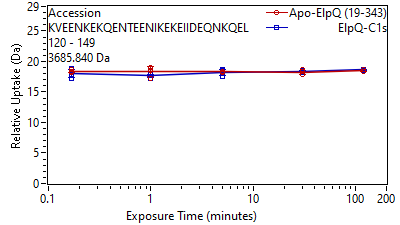

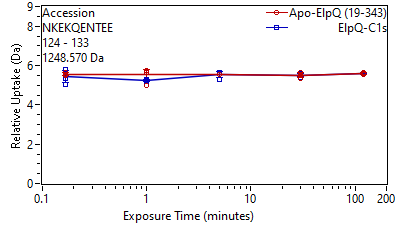

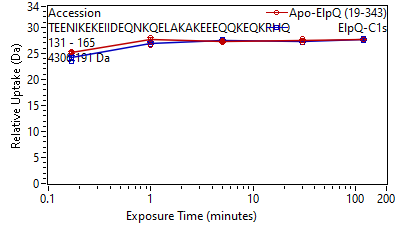

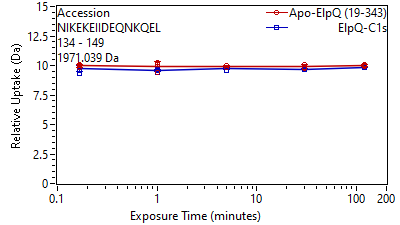

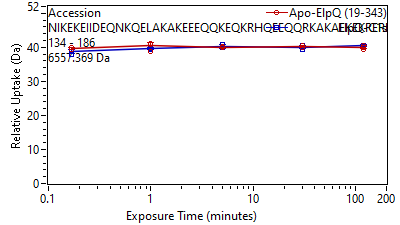

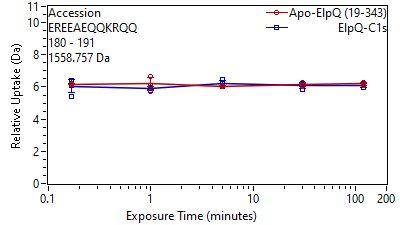

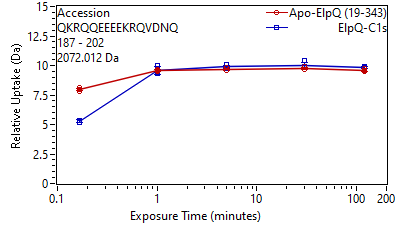

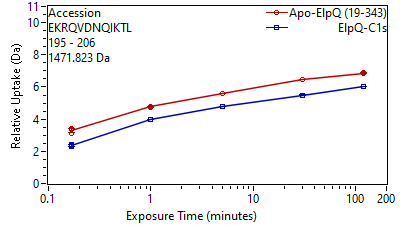

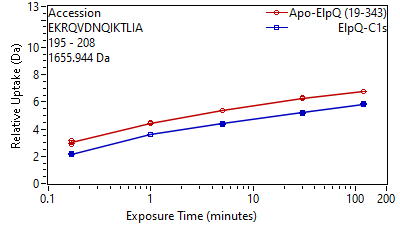

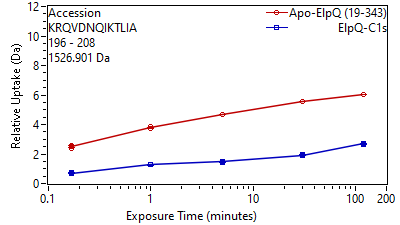

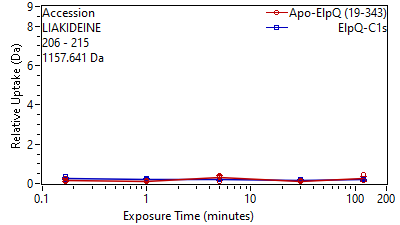

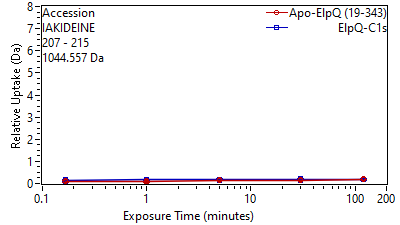

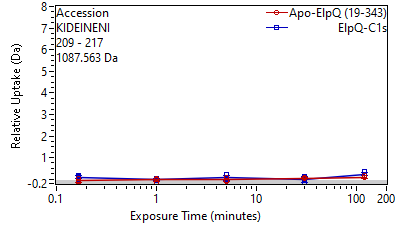

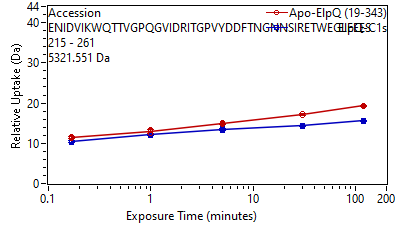

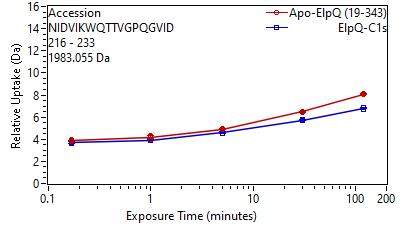

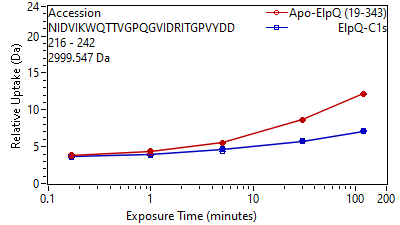

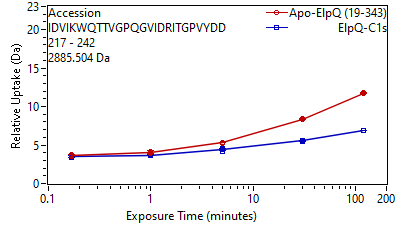

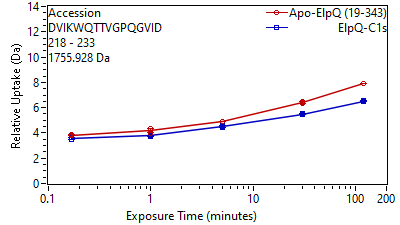

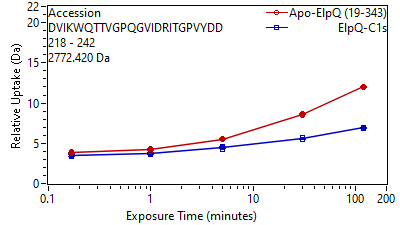

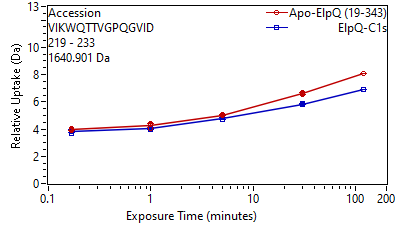

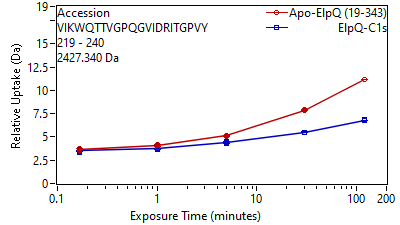

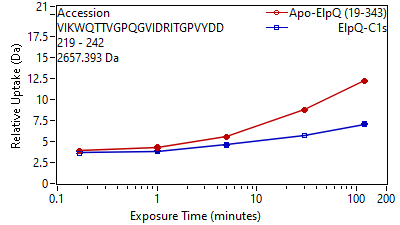

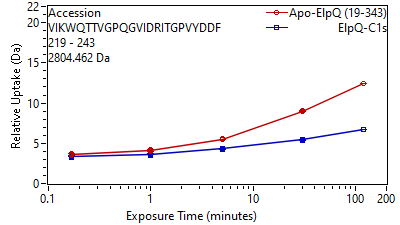

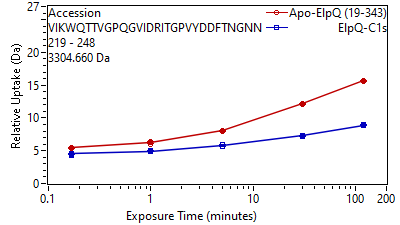

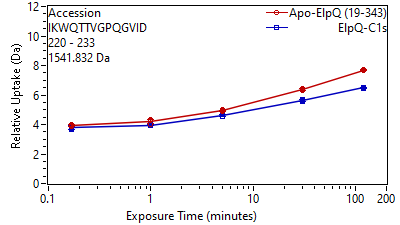

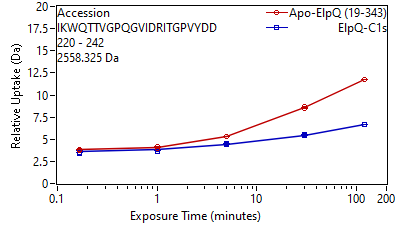

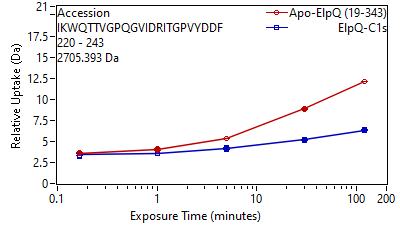

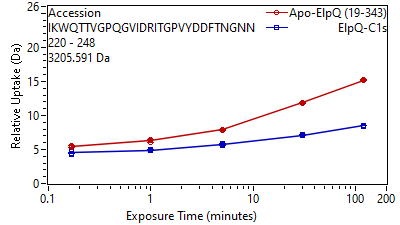

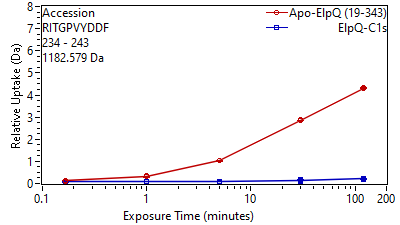

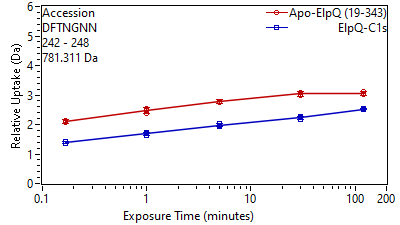

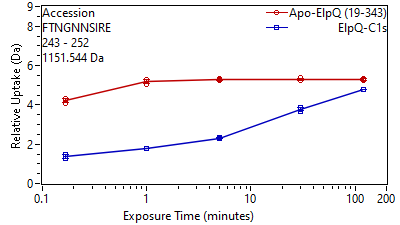

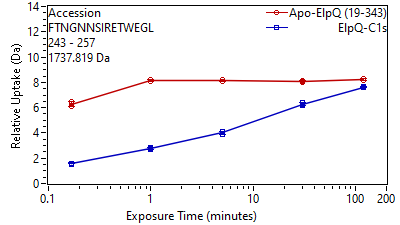

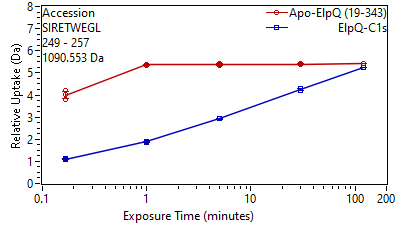

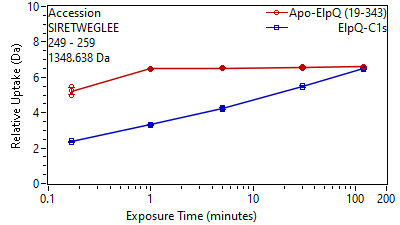

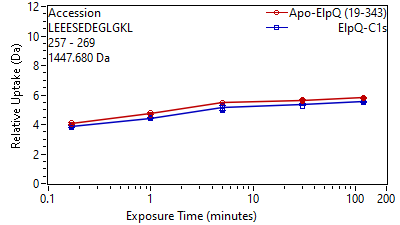

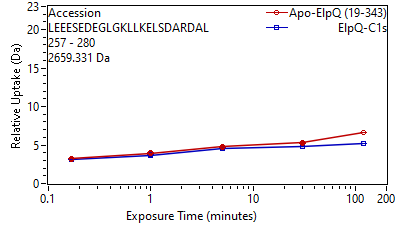

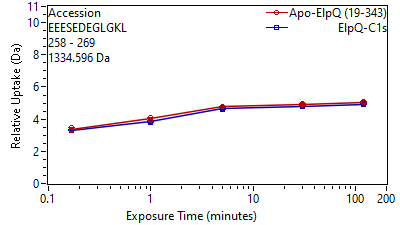

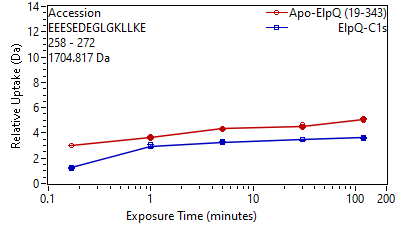

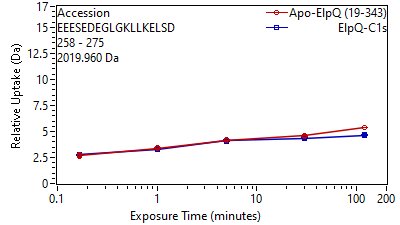

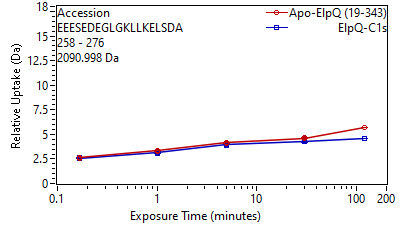

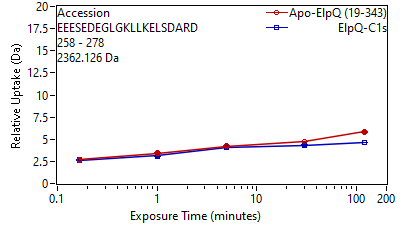

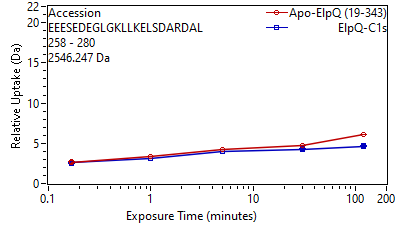

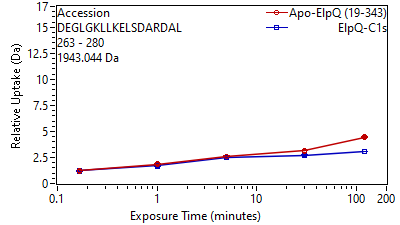

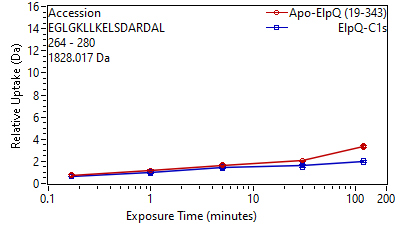

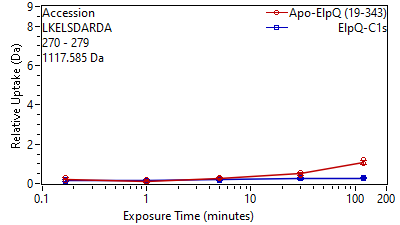

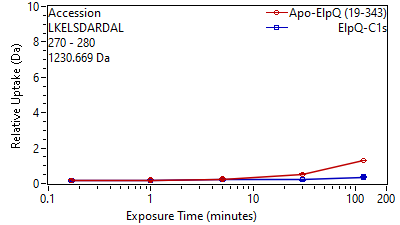

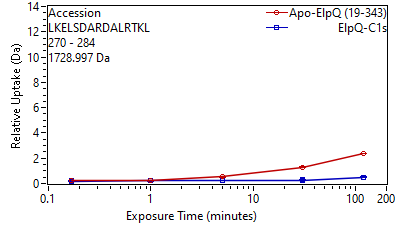

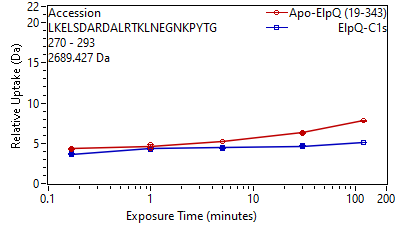

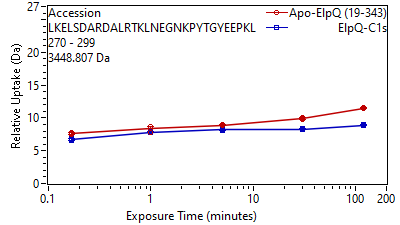

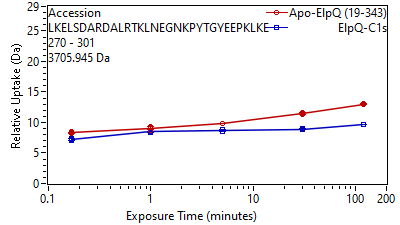

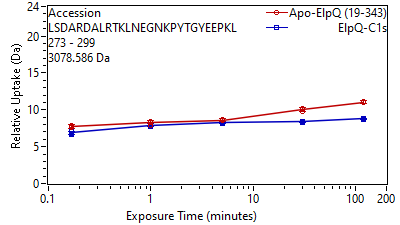

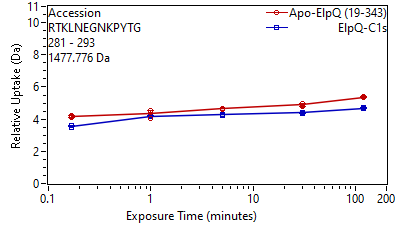

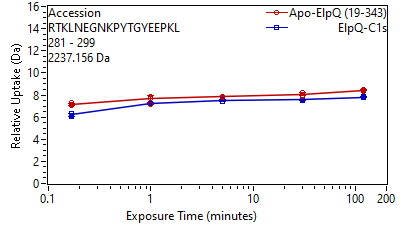

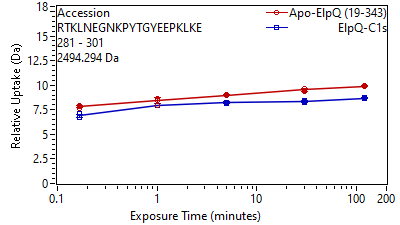

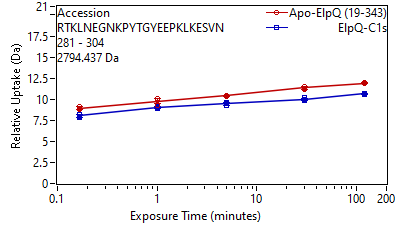

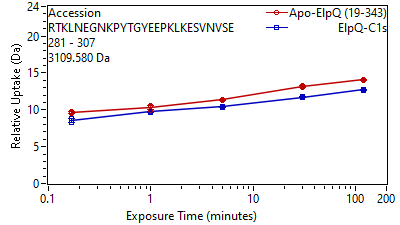

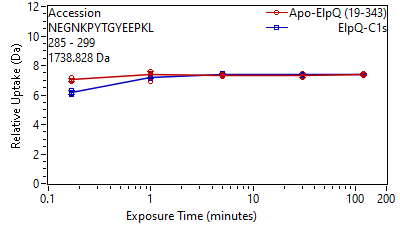

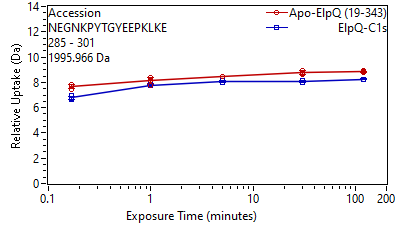

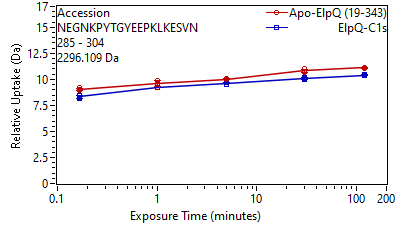

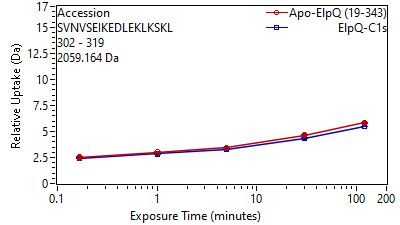

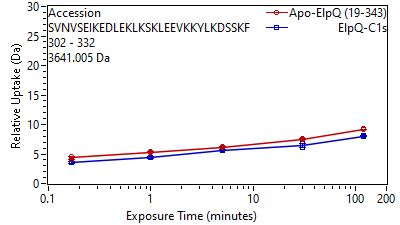

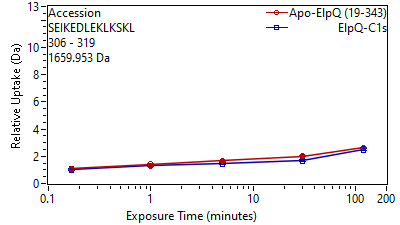


**Figure S1A.** **Peptide coverage map and uptake plots of Apo-ElpQ_19-343_ and ElpQ-C1s HDX-MS experiments.** Apo-ElpQ_19-343_ (red); ElpQ-C1s (blue).

**Figure S1B.** **Spectral plots of Apo-ElpQ_19-343_ and ElpQ-C1s HDX-MS experiments.** Peptides KRQVDNQIKTLIA, RITGPVYDDF, SIRETWEGL, LKELSDARDALRTKLNEGNKPYTGYEEPKLKE, and EEIKGYISDSQ. Arrow points to tics used in the analysis.

**0 s**

**10 s**

**1 min**

**5 min**

**30 min**

**2 h**

**0 s**

**10 s**

**1 min**

**5 min**

**30 min**

**2 h**

**Figure S2.** **Peptide coverage map and uptake plots of Apo-ElpQ_181-343_ HDX-MS experiment.**

**Figure S3. Peptide coverage map and uptake plots of Apo-ElpB_182-378_ HDX-MS experiment.**

**Figure S4.** **AlphaFold2 model of full-length ElpQ.** Coloring is based on pLDDT scores (model confidence) (<https://alphafold.ebi.ac.uk/entry/Q9S035>).

**A.**

Fractional deuterium uptake

(% of max uptake)

77%

0%

**B.**

Fractional deuterium uptake

(% of max uptake)

74%

0%

**Figure S5.** **Fractional deuterium uptake of truncated Apo-ElpQ_181-343_ and Apo-ElpB_182-378_.** **A.** Apo-ElpQ_181-343_, minimum uptake 0% (blue); maximum uptake 77% (red). **B.** Apo-ElpB_182-378_, minimum uptake 0% (blue); maximum uptake 74% (red). Exposure at 30 min. HDX reactions were performed in duplicate (Apo-ElpQ_181-343_ or Apo-ElpB_182-378_). The model of ElpB_182-378_ was created using the full-length model predicted by AlphaFold2 (<https://alphafold.ebi.ac.uk/entry/H7C7R2>).

**B.**

**A.**

**D.**

**C.**

**E.**

**Figure S6. ElpQ and ElpB site-directed mutants retain alpha-helical signature.** Circular dichroism spectra for **A.** ElpQ_Y240A_. **B.** ElpQ_A4_. **C**. ElpQ_Δ290-296_, we note that the y-axis is not in the same range as the other spectra. **D.** ElpQ_181-340_. **E.** ElpB_Y266A_.

**B.**

**A.**

[C1r]

200 nM

[C1s]

200 nM

**Figure S7. ElpB_Y266A_** **SPR binding assays with C1s and C1r.** A two-fold series (0-200 nM) was injected over immobilized ElpB_Y266A_ **A**. C1s. **B.** C1r. Sensorgrams from a representative injection series are shown as black lines and injection series were conducted in triplicate (n = 3).

ElpA1_297 MNKIMKIFIICAVFALINSCKYYSTNKDSKDL--------------KSAKQGLKNRVKGA 46

ElpA2_297 MNKKIKIFIICTVVVLISSCKYYARDKDLKQN--------------------VKEQIGGF 40

Elp26_N40 MNKIIKILIICSVFVLISSCKNYAIK---------------------DLEQKTKGQVNGF 39

ElpX_B31 MNKKMKIFIICAVFVLISSCKIDATGKDATGKDATGKDATGKDATGKNAEQNIKGKVQGF 60

ElpQ_B31 --MNKKTLIICAVFALIISCKNFATGK--------------------DIKQNSEGKIKGF 38

ElpB2_297 --MNKKTFIICAVFALISSCKNFTTSK--------------------DLKQNPEGKIKGF 38

ElpD_B31 --MNQKAFIICAVFALIISCKNYATSK--------------------DLKQNPEGKIKGF 38

Elp22_N40 --MNQKAFIICAVFALIISCKNYATSK--------------------DLKQNPEGKIKGF 38

ElpB_B31 --MNKKTIIICAVFALILSCKNYAI-K--------------------DLEQNAKGKIKGF 37

ElpB1_297 --MNKIMFIICAVFALISSCKNYASSE--------------------DLKQNAKGKIKGF 38

ElpM_B31 --MNKKILIIFAVFALIISCKNYATGK--------------------DIKQNAKGKIKGF 38

Elp24_N40 --MNKKILIIFAVFALIISCKNYATGK--------------------DIKQNAKGKIKGF 38

:** :*..** *** : : :: *

ElpA1_297 LD----ILNIKDEIASSGSKVYELAKEEQKKEKTIVGEIARKLQEEDEAAKDKEDNKQDV 102

ElpA2_297 -------LDTKERIVSDDPTVYGIAEKLKE---------------EE--LKGKKENTKDV 76

Elp26_N40 I--DRALDPTKDKIASNGPTVDKLAKKLQEEE---------K----EELMQGDDPSGIGI 84

ElpX_B31 L--EKILDPVKDKIASNGPIADELAKKLQEEEKVNNGEEEND----KAVFLGEESKEDEE 114

ElpQ_B31 V--NKILDPVKDKIASSGTKVDEVAKKLQEEE---------K----EELMQGDDPNGSGI 83

ElpB2_297 LVNNNILDPTKDKIASSGSKVNEVAKKLQE----------------EELMQGDDPNNGVI 82

ElpD_B31 L------DPTKDKITSIGPKVDEVAKKLKQE----------E----RELMQGDDPNGSGI 78

Elp22_N40 L------DPTKDKITSIGPKVDEVAKKLKQE----------E----RELMQGDDPNGSGI 78

ElpB_B31 I--DKALDPAKDKITSSSSKVDELARKLQEEDKIKGVEENNK----DELMQGDDPNSGVI 91

ElpB1_297 I--DKALDPAKDKITSSSSKVDELARKLQEEDKIKGVEENNK----DELMQGDDPNSGVI 92

ElpM_B31 L--DKVLDPAKDKITSSSSKVDELAKKLQEEDE--------D----NELMQGDDPNNRAI 84

Elp24_N40 L--DKVLDPAKDKITSNGPKVDEVAKKLQEE----------------ELMQGDDPNNSQL 80

*:.*.* . . :*.: :: ... .

ElpA1_297 DLEKKEEPK-NLLKN---DNGL--KPESIA------------------------------ 126

ElpA2_297 NLENKEDSNKNDLKDHEDATVLKPEFEPIA------------------------------ 106

Elp26_N40 N------PP-LVLPENDHDNTPAPKVKAAKQSGGQQEKKVEDQNGEK------------- 124

ElpX_B31 E------------------NEQAVNL---EEKNAEEDKKVVNLEEKE------------- 140

ElpQ_B31 N------PP-PVLPENIHNNALVLKA--IEQSDGQQEKKVEEAE---------------- 118

ElpB2_297 N------PP-SVLSESGQDNAPVSTGKAEEQGGGQQEQKAKEAESKGEEEKVEGKKEKQD 135

ElpD_B31 N------PP-PTLLENGNDNTLVPIAKSAEQSGDQKEEKSGKVEEKK------------- 118

Elp22_N40 N------PP-PTLLENGNDNTLVPIAKSAEQSGDQKEEKSGKVEEKK------------- 118

ElpB_B31 N------SS-PVLPENSQDNT--PILKAAEQSDGQQEEKVKKVEESE------------- 129

ElpB1_297 N------SS-PVLPENSQDNS--PILKAAEQSGGQREVKVKKVEVSE------------- 130

ElpM_B31 A------LL-PVLPENSHDNPPVPKVKAAAQSGGQQEDQKAKESKDK------------- 124

Elp24_N40 S------PP-LVLLASGQDNALVLKAE-QQQSGGQQEEQAKARKEDK------------- 119

.

ElpA1_297 -----SENTEAKLETEKI-----LKPEISERPK---FSVAVKP-EVKEKIEE-------- 164

ElpA2_297 -----LENKEPKLEIAEI-----LKPELPEMPK---LPVVVKP-VVKEKTEE-------- 144

Elp26_N40 ----------KRQEQEEEKVKAKAEKEKSERQKRQ------------------------Q 150

ElpX_B31 -----LEVKK---ETE-----EDEDKEEIEKQK--------------QEVEKA------- 166

ElpQ_B31 ---AKVEENKEKQENTEE---NIKEKEIIDEQNKQELAKAKEEEQQK------------- 159

ElpB2_297 SEKGKVEEKKEKQENAEG---NTKGKEVIEQQKKQQEETAKKAKAQKEKREREQKIQQKQ 192

ElpD_B31 ---EKQESKEEKVEEQNQEKKQK---K----EER-------NVKEEKQKQEEE--RQKQQ 159

Elp22_N40 ---EKQESKEEKVEEQNQEKKQK---K----EER-------NVKEEKQKQEEE--RQKQQ 159

ElpB_B31 ---AKVEGKEEKQENTEERNKQELAKQEEEQQKR-------KAEQEKQKREEEQERQKRE 179

ElpB1_297 ---AKVEGKEEKQENTEVRNKQELAKQEEEQQKR-------KAEKEKQKR---------E 171

ElpM_B31 ---VEEEKEV----VEEKKEEQDSKKEKVEKQS------------QKQKEEERNSKEEQQ 165

Elp24_N40 ---GKGEKAEDRKEKQEDKEGSDKEKEEVEEQN------------KGQKQ---------E 155

: .

ElpA1_297 --EKAKEKEAEELRRR---------RIEQ----Y--KKEEKARLERKKEREIKKKLRELE 207

ElpA2_297 --EKAKDKEYEELKRR---------RIEH----Y--QKQEEERLKRKKQREERKKLRELK 187

Elp26_N40 EEQQRKAKKE------------------------------HQEKEHQEKERER-----ER 175

ElpX_B31 --QERKQR---------------------QEEKKRKKQ---------EQQEEK----KRK 190

ElpQ_B31 --------------EQKR----------HQEEQQRKAKA--EKEKREREEAEQ----QKR 189

ElpB2_297 EEQQRRAKEEEEQQRRAKEEEEQQRRAKEEEEQQRRAKEEEERQRRAKEEEER----QRR 248

ElpD_B31 --EEARAKAE-------------------------------------KEKRER-----EE 175

Elp22_N40 --EEARAKAE-------------------------------------KEKRER-----EE 175

ElpB_B31 EEQERKAKAE----------KEAKEKAE------RQ-K--QEEQQKRKAEKER-----EE 215

ElpB1_297 EVQERKAKAE----------KEAKEKAE------RQ-K--QEEQQKRKAEKER-----EE 207

ElpM_B31 KQEEAKARAD----------REREERLK-QQEQKRQQE--EARVKAEKEKQER-----EE 207

Elp24_N40 AEKEAKE--K----------QEREE------KQKRQQE--EQ-QRKAKEEAEK-----EA 189

. . :

ElpA1_297 SSENFLEGITRSKISTVIKNVDKIISDIDSINLG-SFEEKSEVSGKDVEDKVTGAIYDHI 266

ElpA2_297 SNENYMITLTKGVIFRFSKDIDKIINEINSINLSFSFEEKSEISGKDVEDRVTGAIYDYI 247

Elp26_N40 EREREEEKQVKDKIKDFVDKIDKINRDIDSINPKSFFEERMEVSGQEVEDKVTGAIYDKI 235

ElpX_B31 RQEQRKERRAKNKIKKLADKIDEISWNIDGIESQ------TSVKPKAVIDKITGPVYDYF 244

ElpQ_B31 QQEEEEKRQVDNQIKTLIAKIDEINENIDVIKWQ------TTVGPQGVIDRITGPVYDDF 243

ElpB2_297 AKEEEEKRQVDAQIKRLTSKIDEINGDIDVIKDR------VSVGAEEVRDKITGPVYDYF 302

ElpD_B31 KQKQEEEKKVKGRIKTLTDKINEINRDIDGIKGQ------TSIGAEEVRDKITGPIYDDF 229

Elp22_N40 KQKQEEEKKVKGRIKTLTDKINKINRDIDGIKGQ------TSIGAEEVRDKITGPIYDDF 229

ElpB_B31 QRKEAEKRQVDNEIRTLTGKIDEINRNIDVIKEQ------TSVGAQGVIDRITGPVYDDF 269

ElpB1_297 QRKEAEKRQVDNEIRTLTGKIDEINRNIDVIKEQ------TSVGAQGVIDRITGPVYDDF 261

ElpM_B31 QQKQEEEKKVKYKIKTLTDKIDEINKDIDGINGK------TIVGAEEVIDKITGPVYDDF 261

Elp24_N40 KEKQEEEKKVKDKIKNLVDKIAKINGDIDGIKGK------TSVGAEEVRDKITGPVYDDF 243

:. . * . .: :* :*: *: : : * *::** :** :

ElpA1_297 TNGSSSENSIYSEWGDY---LEEES-GLRSLIEELEEPEEN------------------- 303

ElpA2_297 TDNRSNGNSIYSEWSDY---LEEEENELKSLIEKLEKARTGLRGKIKEVK---------D 295

Elp26_N40 TSDNSRDNSLYSIWDES--IELDESGRLKNLIDDLEKARGELRAKIKEDEYDS------K 287

ElpX_B31 TDDN--KKAIYKTWGDL---EDEEGEGLGKLLKELSDTRDELRTKLNKDNKKYYA---HE 296

ElpQ_B31 TNG---NNSIRETWEGL--EEESEDEGLGKLLKELSDARDALRTKLNEGNKPYTG----Y 294

ElpB2_297 VDG---ENSIRKTWGGGDLEEDDEDSDLGKLLKELSDTRDGLRTKLNEGNKPHTG----D 355

ElpD_B31 TDS---SSSIRTTWGDL---EYEEDLELGRLLKELSEARDSLRTKLNVDNQPYIIDTR-S 282

Elp22_N40 TDS---SSSIRTTWGDL---EYEEDLELGRLLKELSEARDSLRTKLNVDNQPYIIDTR-S 282

ElpB_B31 TDG---NKAIYKTWGDL---EDDNDEGLGKLLKELSDTRHNLRTKLNEGNKAYIIDTR-S 322

ElpB1_297 TDG---NKAIYKTWGDL---EDDNDEGLGKLLKELSDTRHNLRTKLNEGNKAYIIDTR-S 314

ElpM_B31 TDG---NKAIYKTWGDL---EDEEGEELGKLLKELSDTRHNLRTKLNEGNKAYIV---LE 312

Elp24_N40 TDDS--SKAIYYTWN-L---ENEEDSELEKLLKELSDARSSLRTRLNEGNKPLVYTIRKE 297

... .:: * .: * *:..*.. .

ElpA1_297 ------------------------------------------------------------ 303

ElpA2_297 GKERNKNVVGINDIKEDLEKLKDFLIELKEYLQNNVDKKEIHKAVKCSMNPDDVDCE--- 352

Elp26_N40 NNQKNKNIVKVGDIKSDLEKLKSKLDKVKEYPEDKDNFETIKGYIEDSNS---------- 337

ElpX_B31 NEPPLKENVDVSEIKEDLEKVKSGLEKVKEYLKDNSKFEEIKGYISYSQ----------- 345

ElpQ_B31 EEPKLKESVNVSEIKEDLEKLKSKLEEVKKYLKDSSKFEEIKGYISDSQ----------- 343

ElpB2_297 KEPKLKTSVNVSDIKGDLEKLKSYLEKVKGYLENKDNFEDIKGYIEDSNLY--------- 406

ElpD_B31 TEPQLKDNVSVSEIKSDLDELKSKLEEVKEYLEDKDNFEEIKEYVAGSEDNYDEED---- 338

Elp22_N40 TEPQLKDNVSVSEIKSDLDELKSKLEEVKEYLEDKDNFEEIKEYVAGSEDNYGEED---- 338

ElpB_B31 TEPQLKENVSVSEIKSDLDELKSKLEEVKEYLEDKDNFEEIKEYVAGSEDNYDEED---- 378

ElpB1_297 TEPQLKENVSVSEIKSDLDELKSKLEEVKEYLEDKDNFEEIKEYVAGSEDNYDEEDNFRY 374

ElpM_B31 KEPNLKENVNVSDIQSDLEKLKSGLEEVKKYFENEDNFEEIKGYIEDSNSY--------- 363

Elp24_N40 DEPKLKEIVNVSEIKSDLEKLKSKLEDVKKYLEDNSKFEEIKGYITDNSDYYDED----- 352

ElpA1_297 - 303

ElpA2_297 - 352

Elp26_N40 - 337

ElpX_B31 - 345

ElpQ_B31 - 343

ElpB2_297 - 406

ElpD_B31 - 338

Elp22_N40 - 338

ElpB_B31 - 378

ElpB1_297 N 375

ElpM_B31 - 363

Elp24_N40 - 352

**Figure S8. Alignment of Elp proteins and homologs from *B. burgdorferi* strains B31, N40 and 297.** Amino acid sequence alignment of the Elp proteins with their paralogs from strains B31, N40 and 297. Symbols indicate the following: (*) identical, (: or .) similarity, (-) missing residue. Putative C1s-binding sites in ElpQ are highlighted in respective colors, along with corresponding residues found in Elp paralogs. Y240 (yellow); the four glutamates mutated in ElpQ_A4_ (blue); loop residues deleted in ElpQ_Δ290-296_ (purple); last three residues DSQ deleted in ElpQ_181-340_ (green).

| **Data Set** | **Apo-ElpQ_19-343_** | **ElpQ-C1s** |
| --- | --- | --- |
| HDX reaction details | 25 mM Phosphate buffer, pD 7.5, 0.1 M NaCl | |
| HDX time course (min) | 0, 0.167, 1, 5, 30, 120 | |
| # of Peptides | 110 | 110 |
| Sequence coverage | 96% | 96% |
| Average peptide length / Redundancy | 19.1/6.74 | 19.1/6.74 |
| Replicates (biological or technical) | 3 (technical) | 3 (technical) |
| Repeatability | 0.102 (average standard deviation) | 0.098 (average standard deviation) |
| Significant differences in HDX (delta HDX > X D) | ΔHDX < 0.5 Da and *p-*value < 0.01 in a Welch’s *t*-test | |
| **Data Set** | **ElpQ_181-343_** | |
| HDX reaction details | 25 mM Phosphate buffer, pD 7.5, 0.1 M NaCl | |
| HDX time course (s) | 0, 0.167, 1, 5, 30 | |
| # of Peptides | 66 | |
| Sequence coverage | 99% | |
| Average peptide length / Redundancy | 11.8/4.65 | |
| Replicates (biological or technical) | 2 (technical) | |
| Repeatability | 0.044 (average standard deviation) | |
| Significant differences in HDX (delta HDX > X D) | NA | |
| **Data Set** | **ElpB_182-378_** | |
| HDX reaction details | 25 mM Phosphate buffer, pD 7.5, 0.1 M NaCl | |
| HDX time course (s) | 0, 0.167, 1, 5, 30 | |
| # of Peptides | 93 | |
| Sequence coverage | 95% | |
| Average peptide length / Redundancy | 11.8/5.74 | |
| Replicates (biological or technical) | 2 (technical) | |
| Repeatability | 0.043 (average standard deviation) | |
| Significant differences in HDX (delta HDX > X D) | NA | |

**Table S1. HDX data summary.**

NA, not applicable.
